# Organophosphonate Ligation Approach for the Controlled Assembly of Gigantic Polyoxometalate Clusters

**DOI:** 10.1021/jacs.5c21427

**Published:** 2026-03-17

**Authors:** Mengyuan Cheng, Yujia Li, Rongqing Tang, Yubin Ma, De-Liang Long, Leroy Cronin, Weimin Xuan

**Affiliations:** † State Key Laboratory of Advanced Fiber Materials & College of Chemistry and Chemical Engineering, 12475 Donghua University, Shanghai 201620, P. R. China; ‡ School of Chemistry, 3526 University of Glasgow, University Avenue, Glasgow G12 8QQ, U.K.

## Abstract

The controlled assembly of gigantic polyoxometalate (POM) clusters remains one of the most formidable challenges in molecular self-assembly, as it is highly dependent on delicate synthesis parameters that can yield a wide variety of products. In this study, we report the synthesis of a series of unprecedented wheel-shaped molybdenum-blue (MB) clusters directed by organophosphonate (L) and acetate ligands, resulting in a new range of giant MB-type POMs: {Mo_136_Na_4_}, {Mo_120_}, {Mo_118_Na_2_}, {Mo_118_}, and {Mo_157_}. These structures, constructed from fundamental {Mo_1_}, {Mo_2_}, and {Mo_8_} building blocks, exhibit new features of organic ligand coordination on their exterior surfaces. Notably, the {Mo_157_} framework acts as a host capable of capturing the fully reduced ε-Keggin-based {Mo_16_} guest. It represents the first pure dodecameric Mo wheel reported to date. Systematic variation of reaction parameters—including ligand type, concentration, solvent composition, and precursor identity—enabled precise control over cluster topology, revealing competitive coordination between organophosphonate and acetate ligands. Structural analyses unveiled new connection modes involving reduced edge-sharing {e-Mo_2_} units and their derivative {Mo_3_L_2_} motifs, which reinforce the overall cage architecture. Mass spectrometry and NMR spectroscopy confirmed the structural integrity of these assemblies in solution. This work not only expands the library of gigantic MB clusters but also establishes a new strategy for their controlled construction using anchored organophosphonate ligands. The resulting clusters exhibit significantly enhanced solubility in organic solvents compared with traditional MB species, offering new opportunities for postsynthetic modification, improved interactions with biomolecules, and diverse applications.

## Graphical abstract

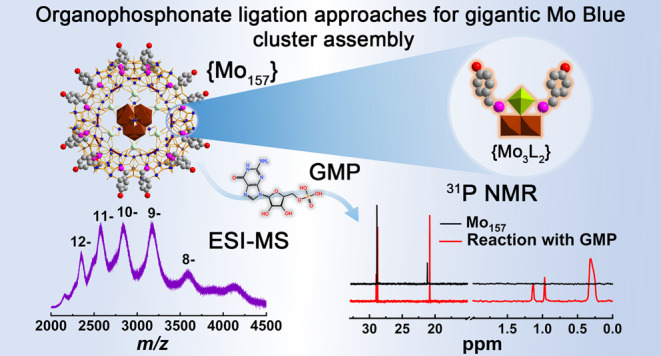

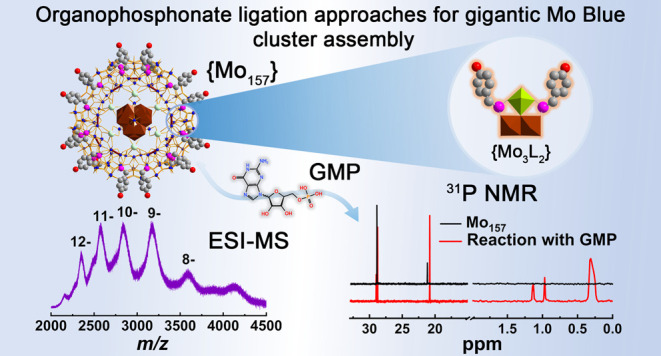

## Introduction

Polyoxometalates (POMs) are a unique class of discrete metal-oxo clusters known for their diverse structures and rich physicochemical properties.^1,2^ Gigantic polyoxomolybdate (POMo) clusters represent an important subclass of POMs, consisting of hundreds of molybdenum centers, and hold great potential for applications in catalysis,^3,4^ sensing,^5^ materials,^6^ and biomedicine.^7^ Based on their degree of reduction and structural building blocks, gigantic POMo clusters are conventionally classified into three types, namely, molybdenum blues (MBs), molybdenum browns (Mo browns), and molybdenum reds (Mo reds), which together give rise to a wide variety of fascinating structures.^8–10^ MBs’ blue coloration is due to the delocalization of their reducing electrons, while the color displayed by Mo browns and Mo reds comes from localized reducing electrons on the edge-sharing {e-Mo_2_} units.^8a^ Structurally, MBs and Mo browns share common pentagonal {Mo_6_} building units, while Mo browns and Mo reds both incorporate reduced {e-Mo_2_} motifs. To date, a variety of remarkable architectures have been successfully designed and synthesized, including the wheel-shaped {Mo_154_}^8c^ and {Mo_176_}^11^ MB, the Keplerate-type K-{Mo_132_}^12^ Mo brown, and the cage-like {Mo_240_}^9c^ Mo red. While these achievements mark a significant breakthrough in POM chemistry, precise control over cluster size, topology, and composition through rational modulation of reduction degree, pH, and ligand coordination remains in the exploratory stage.^13^ Further understanding of the self-assembly mechanism and the roles of various structure-directing agents is essential for enabling the predictable and controllable synthesis of novel gigantic POMo clusters.

Within the MB family, assembled under acidic and reductive conditions, clusters are particularly fascinating due to their dynamic self-assembly behaviors and aesthetically complex architectures. MB wheel clusters are constructed from basic building blocks (BBs), including pentagon-based {Mo_8_}, corner-sharing {c-Mo_2_}, and supporting {s-Mo_1_} units, which are connected in a defined manner to form wheel structures. Generally, the wheel sizes are controlled by the number and type of linker {Mo_2_} units that connect {Mo_8_} BBs (Scheme 1).^14,15^ For example, the number of {c-Mo_2_} units decreases from 16 to 14 as the structure changes from hexadecamer {Mo_176_}^11^ to tetradecamer {Mo_154_}.^8c^ Recent studies have demonstrated that the {c-Mo_2_} units can be replaced with smaller, reduced edge-sharing {e-Mo_2_} units, leading to the formation of decameric capped C-{Mo_132_}^16^ and lantern-shaped L-{Mo_132_}^17^ (Scheme 1). The {e-Mo_2_} is relatively reactive, with its surface-coordinated water molecules readily exchangeable for various ligands, such as sulfate, phosphate, and carboxylate.^18^ We hypothesized that ligand variation on the {e-Mo_2_} unit can create new interaction sites, fascinating the generation of new BBs. Therefore, precise modulation of the number and even structural evolution of {e-Mo_2_} units is very important for the development of novel gigantic MB clusters (Scheme 1).

**Scheme 1:**
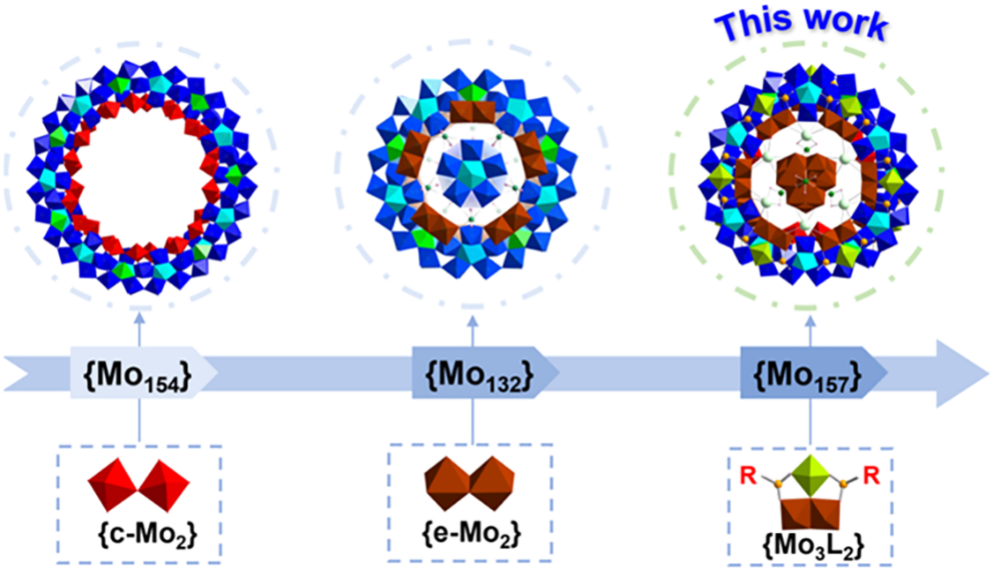
Transformation of {Mo_2_} Linker Units from {c-Mo_2_} to {e-Mo_2_} and Derived {Mo_3_L_2_} Leading to Structural Evolution of Gigantic POMo Clusters, Revealing the Importance of Building Blocks in Structural Assembly^a^

a L represents organophosphonate ligands.

Organophosphonates are versatile ligands for cluster synthesis due to their diverse coordination modes, intermediate ligand field strength, and tunable steric properties.^19^ However, their application in the construction of POMo clusters remains relatively limited.^20,21^ Most reported examples involve low-nuclearity frameworks containing one or a few phosphonate groups. Representative POMo structures include [(Mo_2_^V^O_4_)_4_(O_3_PCH_2_PO_3_)_4_(CO_3_)_2_]^12‑^,^21a^ [Na­(SO_3_)_2_(C_6_H_5_PO_3_)_4_Mo_4_^V^Mo_14_^VI^O_49_]^5–^,^21b^ and [Mo_5_^V^Mo_7_^VI^O_30_(BPO_4_)_2_(C_6_H_5_O_3_P)_6_]^5–^.^21d^ Recent studies have shown that phenylphosphonate ligands can actually replace sulfate or sulfite groups and functionalize the inner surface of gigantic POMo clusters by linking with well-established BBs.^22^ This implies that organophosphonates have the potential to promote the formation of robust coordinate bonds suitable for the assembly of high-nuclearity clusters. Given the abundance of reactive synthons in highly reducing aqueous solutions, we propose that organophosphonates may also interact with {e-Mo_2_} units, thereby triggering the generation of new building blocks. Rational integration of these newly generated and existing BBs will thereby lead to the construction of novel gigantic assemblies with emergent structural and functional properties (Scheme 1).

Herein, we report five novel MB clusters where organophosphonate ligands (L) induce the formation of {e-Mo_2_}-based {Mo_3_L_2_} units: {Mo_136_Na_4_L_18_} (**1**), {Mo_120_L_12_} (**2**), {Mo_118_Na_2_L_20_} (**3**), {Mo_118_L_16_} (**4**), and {Mo_157_L_18_} (**5**) (Figure 1 and Figures S2–S4). Compounds **1**–**4** possess a decameric wheel framework built from pentagon-based {Mo_8_}, {Mo_1_}, and {Mo_3_L_2_} (incorporating variant {Mo_2_NaL_2_}) units. In contrast, compound **5** forms a dodecameric wheel assembled from {Mo_8_}, {Mo_1_}, {Mo_3_L_2_}, and {c-Mo_2_} linkers, with a central fully reduced ε-Keggin {Mo_12_^V^}-based {Mo_16_} guest encapsulated within the hollow interior (Figure 1e). In particular, the {Mo_3_L_2_} BB consists of an {e-Mo_2_} dimer augmented by a growing {g-Mo_1_} unit joined by two organophosphonate ligands. Structural analyses unveil a series of new connection modes involving {e-Mo_2_} and {Mo_3_L_2_} units, which reinforce the overall structural architecture. Mass spectrometry and NMR spectroscopy confirm the structural integrity of these assemblies in solution. These compounds have been comprehensively characterized as detailed in the Supporting Information (Figures S13–17, Tables S4 and S5) with formulas:

$${\left({\mathrm{NH}}_{4}\right)}_{40}H_{16}\left[H_{10}{\mathrm{Mo}}_{136}{\mathrm{Na}}_{4}{\left(H_{2}O\right)}_{16}O_{390}{\left({\mathrm{CH}}_{3}\mathrm{COO}\right)}_{10}{\left({\mathrm{PO}}_{3}C_{7}H_{7}\right)}_{18}\right]\cdot \mathrm{ca}.210H_{2}O\cdot {\left({\mathrm{CH}}_{3}\mathrm{COOH}\right)}_{7}\;1\equiv {\left({\mathrm{NH}}_{4}\right)}_{40}H_{16}\{1a\}\cdot \mathrm{ca}.210H_{2}O\cdot {\left({\mathrm{CH}}_{3}\mathrm{COOH}\right)}_{7}$$

$${\left({\mathrm{NH}}_{4}\right)}_{20}H_{22}\left[H_{10}{\mathrm{Mo}}_{120}{\left(H_{2}O\right)}_{19}O_{344}{\left({\mathrm{CH}}_{3}\mathrm{COO}\right)}_{8}{\left({\mathrm{PO}}_{3}C_{7}H_{7}\right)}_{12}\right]\cdot \mathrm{ca}.130H_{2}O\;2\equiv {\left({\mathrm{NH}}_{4}\right)}_{20}H_{22}\{2a\}\cdot \mathrm{ca}.130H_{2}O$$

$${\left({\mathrm{NH}}_{4}\right)}_{24}H_{12}\left[H_{10}{\mathrm{Mo}}_{118}{\mathrm{Na}}_{2}{\left(H_{2}O\right)}_{18}O_{338}{\left({\mathrm{PO}}_{3}C_{7}H_{6}{\mathrm{OCH}}_{3}\right)}_{20}\right]\cdot \mathrm{ca}.155H_{2}O\cdot {\left({\mathrm{CH}}_{3}\mathrm{COOH}\right)}_{10}\;3\equiv {\left({\mathrm{NH}}_{4}\right)}_{24}H_{12}\left\{3a\right\}\cdot \mathrm{ca}.155H_{2}O\cdot {\left({\mathrm{CH}}_{3}\mathrm{COOH}\right)}_{10}$$

$${\left({\mathrm{NH}}_{4}\right)}_{24}H_{6}\left[H_{10}{\mathrm{Mo}}_{118}{\left(H_{2}O\right)}_{18}O_{336}{\left({\mathrm{CH}}_{3}\mathrm{COO}\right)}_{4}{\left({\mathrm{PO}}_{3}C_{7}H_{6}{\mathrm{OCH}}_{3}\right)}_{16}\right]\cdot \mathrm{ca}.130H_{2}O\;4\equiv {\left({\mathrm{NH}}_{4}\right)}_{24}H_{6}\left\{4a\right\}\cdot \mathrm{ca}.130H_{2}O$$

$$K_{27}{\mathrm{Na}}_{5}H_{4}\left[H_{24}{\mathrm{Mo}}_{157}{\left(H_{2}O\right)}_{35}O_{456}{\left({\mathrm{PO}}_{3}C_{7}H_{6}\mathrm{OH}\right)}_{18}\right]\cdot \mathrm{ca}.240H_{2}O\;5\equiv K_{27}{\mathrm{Na}}_{5}H_{4}\left\{5a\right\}\cdot \mathrm{ca}.240H_{2}O$$

**Figure 1:**
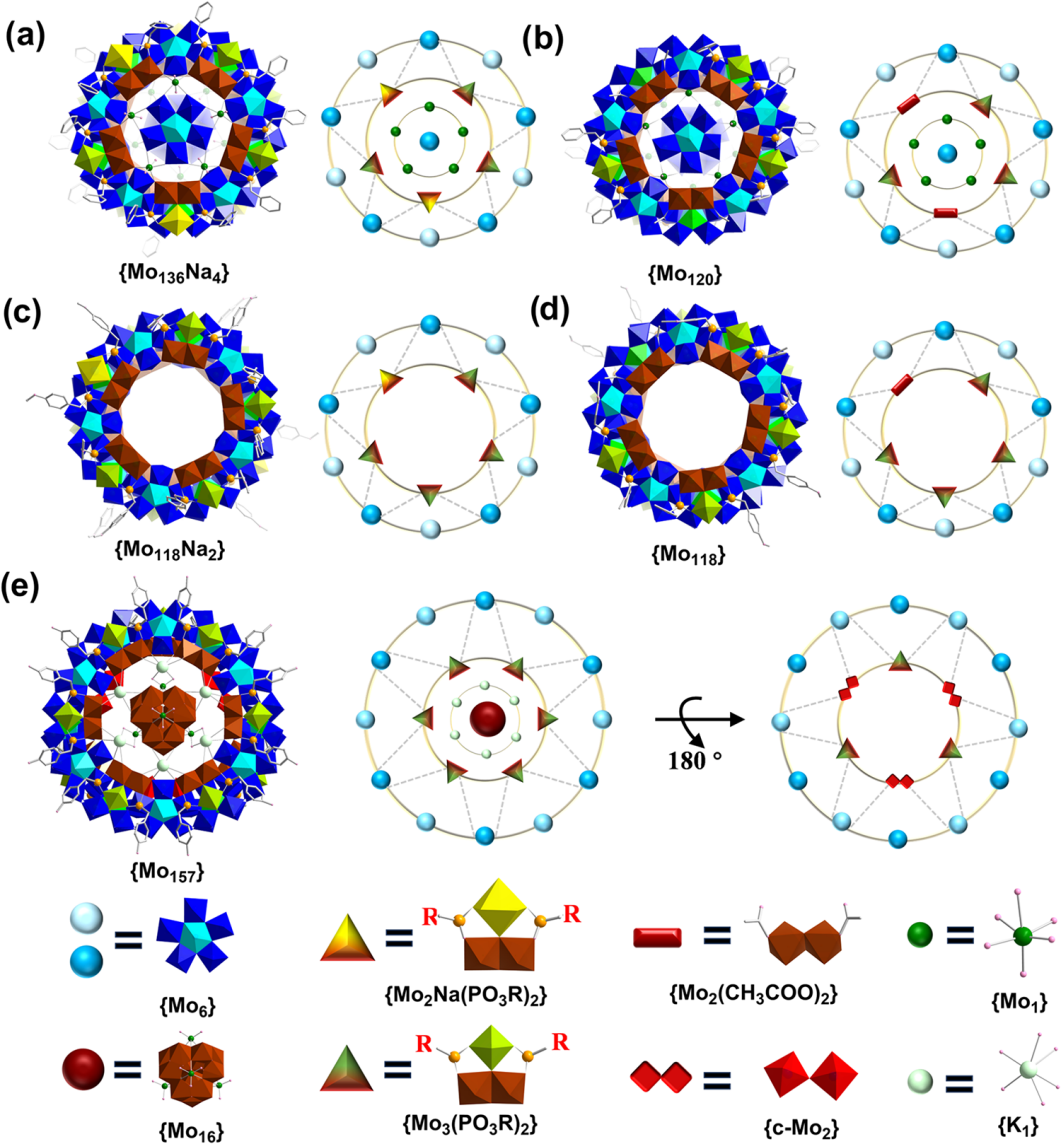
Structural evolution of MB wheels directed by different ligand systems. (a–e) View of the molecular structure of **1a**–**5a** (left) and corresponding simplified connection models (right) with building units defined at the bottom. {s-Mo_1_}, bright green polyhedron; {e-Mo_2_}, brown polyhedron; {c-Mo_2_}, red polyhedron; {Mo_6_}, blue polyhedron pentagonal units with cyan polyhedron center; {g-Mo_1_}, lime polyhedron; {Mo_1_} linker, green ball; K, light green ball; Na, yellow polyhedron; O, rose ball; C, gray ball; and P, light orange ball. Bottom: simplified schematic symbols used in the connection models and their represented structural building blocks ({Mo_16_}, {Mo_2_}, {Mo_6_}, {Mo_3_}, etc.).

## Results and Discussion

Compound **1** was successfully synthesized via the hydrothermal reaction of an aqueous mixture of (NH_4_)_6_Mo_7_O_24_·4H_2_O (0.4 mmol), N_2_H_4_·2HCl (0.5 mmol), benzylphosphonic acid (0.4 mmol), CH_3_COONa (0.5 mmol), 1 mL of acetic acid, and a small amount of HCl in a capped vial at 120 °C for 3 days, which generally favored the formation of the desired framework (Figure S1). Under these highly reducing conditions, the formation of a cyclic cluster with a high Mo­(V)/Mo­(total) ratio is promoted. Meanwhile, the strong coordination of the organophosphonate to {e-Mo_2_^V^} unit not only stabilizes this fully reduced species but also triggers the generation of the {Mo_3_L_2_} motif. The synergistic interplay of these two critical factors facilitates the assembly of the giant Mo-blue wheel during ligand functionalization. Compounds **2**–**5** were formed by varying the amounts of ligands, acid content (pH value), and source of molybdates under similar conditions. The dominant factor lies in alteration of the type of organophosphonates, resulting in benzyphosphonate-functionalized close-shell **1** and **2**, methoxybenzyl phosphonate-functionalized open-shell **3** and **4**, as well as hydroxybenzyl phosphonate-functionalized **5** featuring a cluster@cluster architecture. Moreover, the cation also plays an important role, as **5** can only be obtained when a potassium ion is adopted for the assembly.

Single-crystal X-ray structural analysis reveals that **1** crystallizes in the monoclinic system with centrosymmetric space group *C*2/*m* (Table S1), exhibiting a remarkable closed-shell ellipsoidal architecture (Figure 1a). As depicted in Figure 2, ellipsoidal cluster {Mo_136_} **1a** is assembled from multiple pentagon-based {Mo_8_} and various bridging/supporting units. It is organized into two centrosymmetrically related hemispheres of a {Mo_63_}-type configuration. Each {Mo_63_} hemisphere is composed of a {Mo_52_} ring and an additional pentagon-centered {Mo_11_} capping fragment. The {Mo_52_} ring is built from three {Mo_3_}, two {Mo_2_Na}, and five {Mo_8_} units. The {e-Mo_2_} components from the three {Mo_3_} and two {Mo_2_Na} units are positioned along the rim of the {Mo_52_} inner ring and are referred to as rim-{e-Mo_2_} units (Figure S7). Additionally, five other {e-Mo_2_} units are formed between the {Mo_52_} ring and the {Mo_11_} capping fragment. Each of these beam-{e-Mo_2_} units comprises one Mo atom from the pentagon apex of a {Mo_8_} unit on the {Mo_52_} ring and a Mo atom from the {Mo_11_} cap. These beam-{e-Mo_2_} units function as structural “beams”, extending outward from the main body of the {Mo_52_} ring to support the {Mo_11_} cap. The rim-{e-Mo_2_} units feature a Mo···Mo distance of ca. 2.55 Å, significantly shorter than that in the corner-sharing counterparts {c-Mo_2_} observed in conventional MB wheels, leading to a more compact wheel diameter. The beam-{e-Mo_2_} has a Mo···Mo distance of ca. 2.62 Å, effectively bridging the {Mo_52_} ring and the {Mo_11_} cap. Due to the structural differences between the {Mo_3_} and {Mo_2_Na} units, the {Mo_63_} hemisphere deviates from ideal C_5v_ symmetry and instead adopts *C*_*s*_ symmetry, with two distinguishable head and tail ends. Two such {Mo_63_} hemispheres are joined in a head-to-tail fashion via 10 {s-Mo_1_} supporting units inside the ellipsoid equator, resembling the structural motif observed in C-{Mo_132_}.^16^

**Figure 2:**
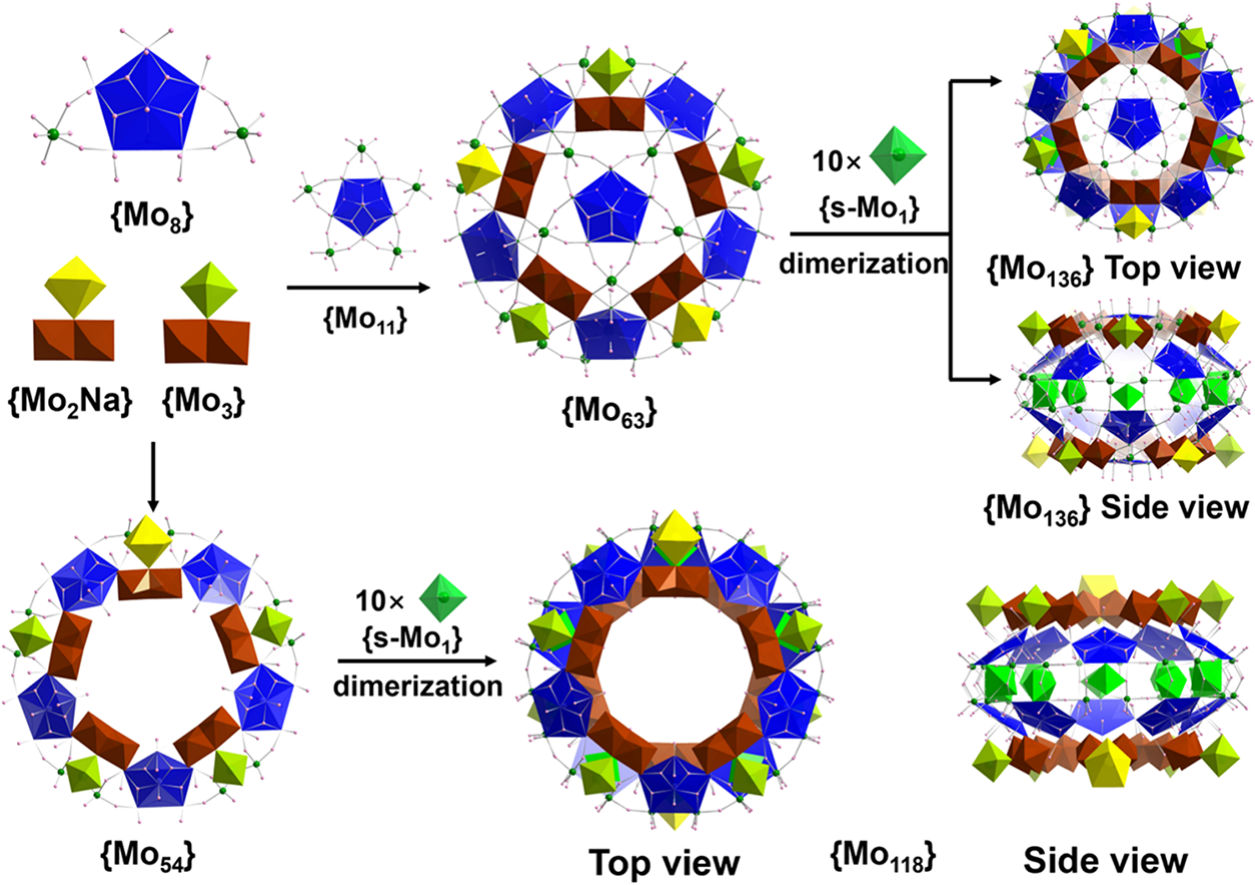
Schematic representation of the construction of {Mo_136_} **1a** and {Mo_118_} **3a** from the pentagon-based {Mo_8_}, {Mo_2_Na}, and {Mo_3_} building blocks with the aid of the supporting units {s-Mo_1_}. The {Mo_63_} features a central ring capped by a {Mo_11_} moiety. The color scheme is the same as that in Figure 1. Organic ligands are omitted for clarity.

In MB wheel systems, ligands readily replace the coordinated water molecules located on the inner surface of {c-Mo_2_} units,^23^ thereby enabling effective stabilization and functionalization of the framework. In **1a**, both bidentate acetate and tridentate phosphonate ligands substitute the water ligands bound to Mo sites on the {e-Mo_2_} unit, distributing over the inner and outer surfaces of the wheel. Specifically, ten acetate ligands are attached to the beam-{e-Mo_2_} units from the inner side, while tridentate organophosphonate ligands bind to the rim-{e-Mo_2_} units (Figure S8). The latter further capture either an additional Mo atom, giving rise to a novel {Mo_3_L_2_} building block, or a Na atom, resulting in a {Mo_2_Na} unit (Figure S9). Through bidentate and tridentate coordination modes, acetate and organophosphonate ligands serve as “molecular nails,” reinforcing the rigidity of the entire cage-like architecture. The incorporation of edge-sharing {e-Mo_2_} units and their derivatives {Mo_3_} and {Mo_2_Na} not only tighten the framework but also differentiates it from previously reported systems employing corner-sharing {c-Mo_2_} linkers. This introduces a new structural motif for designing high-nuclearity molybdenum-oxide clusters with tunable internal cavities. Moreover, the incorporation of organophosphonate ligands significantly improves the solubility of these clusters in a variety of organic solvents (Figure S25), opening avenues for postsynthetic modification and enhanced compatibility with functional materials.

**2** crystallizes in the monoclinic space group *C*2*/m* (Table S1). Unlike **1a**, **2a** {Mo_120_} displays a markedly reduced incorporation of the {Mo_11_} capping unit with an occupancy of ∼ 0.2. Moreover, the higher concentration of acetic acid used in the synthesis of **2a** induces a competitive ligand substitution, whereby acetate ligands replace the phosphonate groups at the corresponding {Mo_2_Na} sites in **1a**. This transformation yields {Mo_2_(CH_3_CO_2_)_2_} units and eliminates the involvement of Na (Figure 1b). These observations reveal that subtle variations in acetic acid concentration not only influence the incorporation of large building blocks but also govern the competitive coordination dynamics between acetate and organophosphonate ligands. Such modulation enables fine-tuning of the final cluster architecture and enhances the structural stability.

**3** and **4** crystallize in the monoclinic space group *P*2_1_/*n* and *C*2/*m*, respectively (Table S2). **3a** and **4a** adopt a decameric wheel structure composed of two {Mo_54_} hemispheres joined by 10 {s-Mo_1_} units. The {Mo_54_} hemisphere in **3a** is built from four {Mo_3_}, one {Mo_2_Na}, and five {Mo_8_} units (Figure 2). The structural difference between **3a** and **4a** resembles that observed between **1a** and **2a**: upon increasing the amount of acetic acid in the reaction mixture, acetate ligands replace the phosphonate groups bound to the {Mo_2_Na} units, resulting in the formation of the {Mo_2_(CH_3_CO_2_)_2_} unit in **4a** (Figure 1c,d). As a result, **4a** represents the acetate-substituted analogue of **3a**, once again highlighting the competitive coordination between the acetate and organophosphonate ligands at the {Mo_2_Na} sites.

Following systematic optimization of the synthetic conditions, compound **5** was successfully obtained in a good yield. Single-crystal X-ray structural analysis reveals that it crystallizes in the monoclinic space group *P*2*/n* (Table S3). The structure of **5a** can be described as a dodecameric wheel composed of two nonequivalent hemispherical shells, top {Mo_82_} and bottom {Mo_63_}, joined by 12 {s-Mo_1_} supporting units, each located behind a pentagonal unit inside the equatorial backbone (Figures 3 and S4), thereby constructing the first pure dodecameric Mo wheel reported to date. The top {Mo_82_} hemisphere is constructed from six {Mo_8_} and six {Mo_3_} building blocks arranged alternately to delineate an elliptical cavity that encapsulates a central ε-Keggin-based {Mo_16_} guest (Figure S10). This {Mo_16_} guest is composed of a fully reduced ε-Keggin core {Mo_12_^V^} and four {Mo^VI^} add-on units and represents the first instance of such a guest being captured in MB synthesis. Several potassium ions are also located between the {Mo_16_} guest and the surrounding wheel framework. Their ionic radius matches the cavity of the dodecameric wheel remarkably well, allowing them to serve a dual role: they not only provide structural reinforcement for the host framework but also anchor the encapsulated {Mo_16_} guest by acting as electrostatic linkers between the host and the guest. In contrast, the bottom {Mo_63_} hemisphere, although also constructed from six {Mo_8_} units, features a distinct linkage pattern in which three {Mo_3_} and three {c-Mo_2_} units are alternatively arranged along the inner rim of the ring (Figure 4). This structural asymmetry between the two hemispheres is a defining characteristic, giving rise to the unique elliptical wheel architecture of {Mo_157_}. The organophosphates on two adjacent {Mo_157_} clusters are interconnected by π–π stacking (centroid-to-centroid distance of approximately 3.9 Å) to form a dimer (Figure S11). Consistent with **1a**–**4a**, 18 benzylphosphonate ligands were found on the exterior surface of **5a**, bridging the {Mo_6_} pentagons and {Mo_3_} units to reinforce the overall wheel structure.

**Figure 3:**
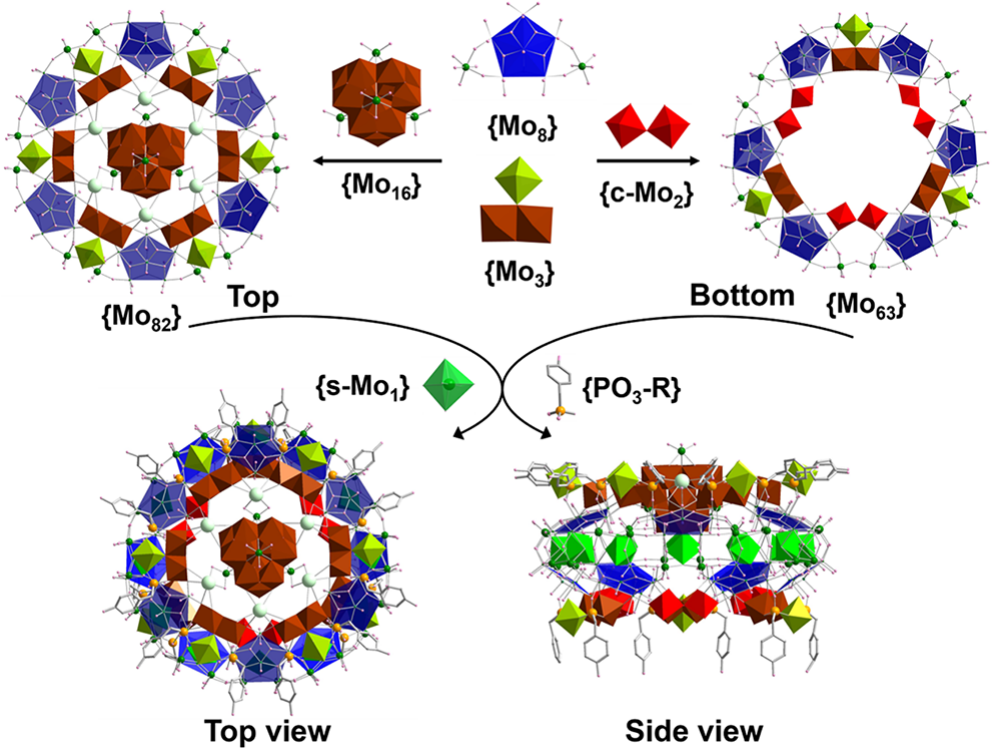
Schematic representation of the construction of {Mo_157_} **5a** from the pentagon-based {Mo_8_} and {Mo_3_} building blocks with the aid of the bridging units {c-Mo_2_}, where the top {Mo_82_} unit encapsulates a central ε-Keggin-based {Mo_16_} guest and connects to the bottom {Mo_63_} through 12 {s-Mo_1_} linkers along the equatorial backbone. The color scheme is the same as Figure 1.

**Figure 4:**
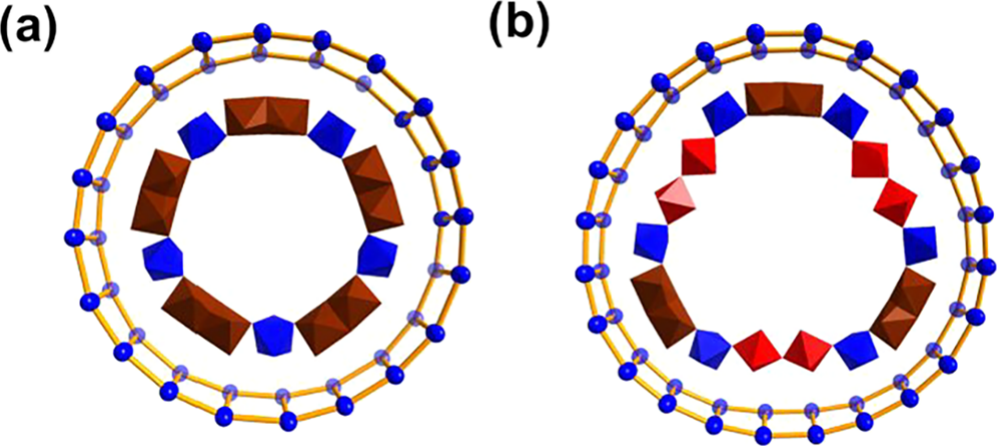
(a) Schematic representation of **4a** and **5a** by connecting Mo atoms on the rim and equator of the wheels. {e-Mo_2_} and {c-Mo_2_} units on the inner rim are highlighted in dark red and red polyhedra.

Compared to traditional MBs, new clusters bearing organophosphonate ligands show greatly improved solubility in polar organic solvents including isopropanol, acetonitrile, and N,N-dimethylformamide, though they remain largely insoluble in dichloromethane (Figure S25 and Table S6). This gives rise to the opportunity to carry out mass spectrum analysis directly in organic media. Electrospray ionization-mass spectrometry (ESI-MS) was employed to study the solution behavior of compounds **4** and **5**. Despite the broad peak envelopes observed in the spectra, a common phenomenon in ESI-MS of giant POMs caused by association of counterions and solvent molecules,^24^ the intact molecular species of **4a** based on the cluster {Mo_118_O_354_H_46_(CH_3_COO)_4_(PO_3_C_7_H_6_OCH_3_)_16_} were clearly detected at charge states ranging from −7 to −10 (Table S7). These signals arise from adding different numbers of ammonium counterions and associated water molecules (Figures 5 and S26). Similarly, for **5**, all major peaks in the spectrum could be assigned to species based on the cluster formula {Mo_157_O_456_H_24_(H_2_O)_35_(PO_3_C_7_H_8_OH)_18_} (Tables S8), with charge states ranging from −8 to −12 (Figures 5 and S27). These findings confirm the presence and stability of **4** and **5** in solution.

**Figure 5:**
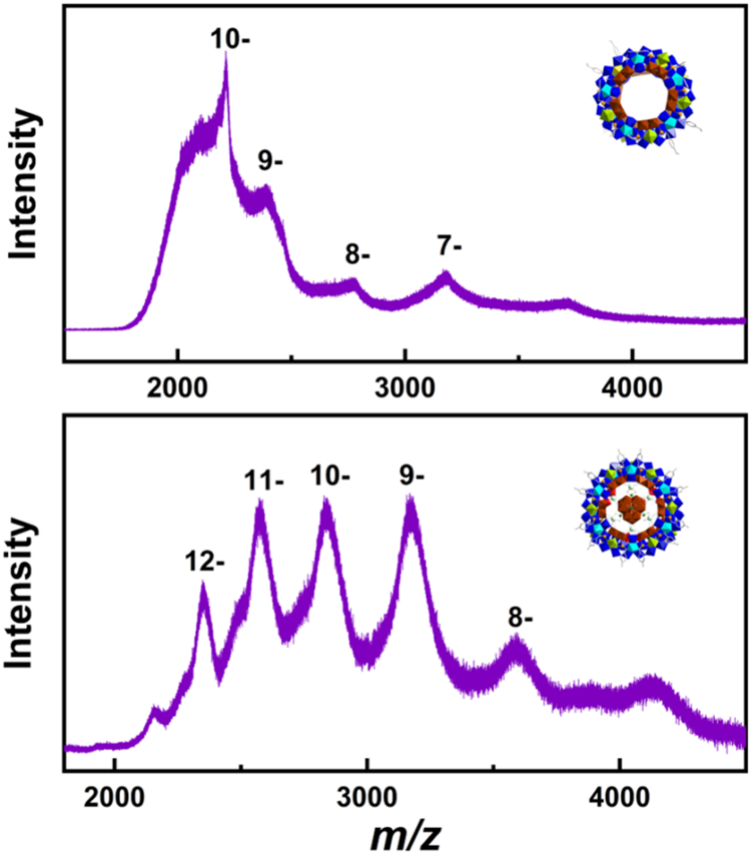
ESI-MS spectra of **4** and **5** in a CH_3_CN solution.

To gain detailed insight into the local environment of the phosphorus center within the compound, we examined the ^31^P NMR spectrum of compound **5**. The spectrum (Figure 6) exhibits two distinct resonances at δ = 21.2 and 28.9 ppm with an intensity ratio of approximately 1:2. This contrasts with the single resonance observed for the free ligand at δ = 25.45 ppm, indicating that the phosphonate ligands experience two chemically nonequivalent environments upon incorporation into the cluster framework. Structurally, the phosphorus atom in the −PO_3_CH_2_C_6_H_4_OH ligand is bonded to three oxygen atoms in an approximately trigonal geometry. These oxygen atoms, however, coordinate to three Mo centers with different oxidation states (two Mo^VI^ and one Mo^V^, Figure 6b). This electronic and structural asymmetry leads to the formation of three conformational isomers of the −PO_3_CH_2_C_6_H_4_OH ligand, arising from rotation around the P–C single bond. These isomers correspond to the 4-hydroxyphenyl (PhOH) group occupying either a trans (1 case) or cis (2 cases) orientation relative to the unique O–Mo^V^ site (Figure 6b), consistent with the observed 1:2 resonance ratio in the NMR spectrum. In the crystal structure, it was found that the trans conformation positions the PhOH group perpendicular to the cluster’s main axis, whereas the cis conformation aligns the PhOH group parallel to the axis (Figure S12). In the solid state, the crystal packing force arising from supramolecular interactions may overcome the energy barrier between these conformational isomers, favoring one dominant orientation. This is exemplified in the crystal structure of {Mo_157_}, where all PhOH groups are oriented parallel to the main axis at one end of the cluster and all perpendicular at the other (Figure S12). In solution, however, it is believed that the three conformational isomers coexist with equal probability, resulting in a theoretical 1:2 ratio as observed.

**Figure 6:**
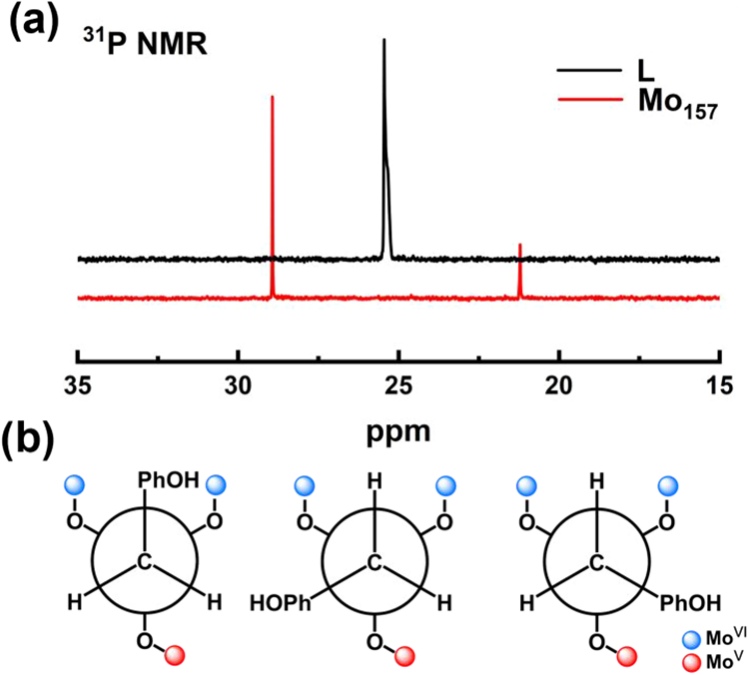
(a) ^31^P NMR spectra of {Mo_157_} and hydroxybenzyl phosphonic acid in D_2_O. (b) Schematic representation of the three possible conformational isomers of the benzylphosphonate ligand arising from rotation around the P–C single bond. The −PO_3_ group is bound to three Mo centers, including two Mo^VI^ (blue) and one Mo^V^ (red). These isomers correspond to the 4-hydroxyphenyl (PhOH) group occupying either a trans (the first case) or cis (the latter two cases) orientation relative to the unique O–Mo^V^ site.

Given the presence of three sets of {c-Mo_2_} units in {Mo_157_} and the known lability of the two coordinated water molecules at the {Mo_2_} sites, we hypothesized that a third type of comparable ligand such as phosphate could substitute at these positions. To explore the potential of postsynthetic modification of the cluster’s exterior microenvironment and test the interaction with biomolecules, we conducted a ligand substitution reaction using guanosine monophosphate (GMP) as a model system (Figure 7). When **5a** was mixed with GMP in D_2_O for 1 h at ambient temperature, the ^31^P NMR spectrum revealed splitting of original organophosphonate peak at 28.9 ppm into two distinct signals at δ 28.98 and 28.76 ppm. Concurrently, the signal at δ 20.81 ppm (originally 21.2 ppm) increased in relative intensity. At the same time, new phosphate resonances appeared at δ 0.96 and 1.12 ppm, characteristic of monoester phosphate environments derived from GMP. These are distinct from the signal at δ 0.31 ppm, which is assigned to free GMP.^25^ Collectively, these spectral changes support the occurrence of ligand substitution at cluster sites adjacent to the bound organophosphonate groups. The ^31^P NMR data confirm that nucleotides such as GMP can successfully replace surface-coordinated water molecules, enabling controlled functionalization of the MB cluster. The resulting Mo–O–PO_3_–nucleotide linkage provides clear spectral markers and establishes a practical starting point for subsequent probe reactions, including simple site-selective labeling strategies.

**Figure 7:**
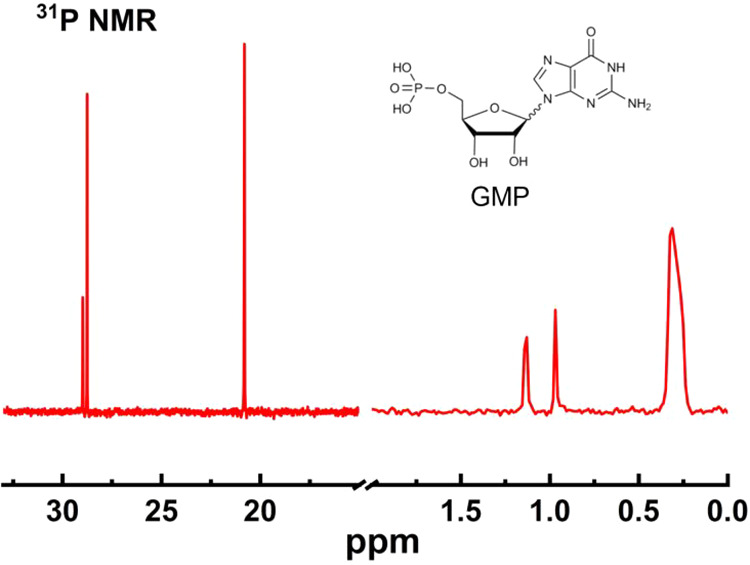
^31^P NMR spectrum of the solution after GMP ligand reacts with {Mo_157_}, showing two new signals around 1.0 ppm, corresponding to coordinated phosphate sites of GMP on the cluster.

## Conclusion

In summary, we have successfully achieved the controlled assembly of five novel molybdenum-blue wheel clusters through the use of semirigid organophosphonate ligands. This study demonstrates that subtle changes in ligand identity and reaction conditions can drastically alter the resulting nuclearity and topology, highlighting the delicate balance between competitive and cooperative interactions among inorganic precursors, organophosphonates, and auxiliary solvents. The introduction of new building blocks—particularly {Mo_3_L_2_} motifs—provides a structural foundation for engineering high-order architectures with tunable cavity sizes and framework rigidity. This work broadens the structural diversity of polyoxomolybdate chemistry and offers valuable insights into the rational design of gigantic POMs with controllable geometry and emerging functionalities. The introduction of functional phosphonate ligands also provides a new avenue for the development of POM clusters as promising platforms for biosensing application.

## Supplementary Material



## Notes

Synthetic procedure, single-crystal structure analyses, formula determination, additional structural figures, thermogravimetric analysis, UV–vis, FTIR spectra and Raman spectra, ESI-MS and NMR, as well as the solubility of compounds **1** and **5** (PDF)
