# Supplementary material for: Organophosphonate Ligation Approach for the Controlled Assembly of Gigantic Polyoxometalate Clusters
Source: J Am Chem Soc. 2026 Mar 17;148(12):12890–7. doi: 10.1021/jacs.5c21427 (PMC13047529; doi:10.1021/jacs.5c21427)
Supplement: Supplementary file 1 [file ja5c21427_si_001.pdf]

Supporting information

Organophosphonate ligation approach for the controlled assembly of gigantic polyoxometalate clusters

Mengyuan Cheng,[a,b] Yujia Li,[a] Rongqing Tang,[a] Yubin Ma,[a] De-Liang Long,[b]* Leroy Cronin[b]*and Weimin Xuan*[a]

[a] State Key Laboratory of Advanced Fiber Materials & College of Chemistry and Chemical Engineering, Donghua University, Shanghai 201620, P R China.

[b] School of Chemistry, University of Glasgow, Glasgow G12 8QQ, United Kingdom.

*e-mail: weiminxuan@dhu.edu.cn; deliang.long@glasgow.ac.uk; lee.cronin@glasgow.ac.uk

Table of Contents

1. Materials 3

2. Instrumentation 3

3. Synthetic procedures for compounds 1-5 4

4. Single-crystal X-ray diffraction analyses 6

5. Crystallographic data and crystal structures of 1-5 10

6. Formula determination 12

7. Structural descriptions 18

8. Spectral characterization of 1-5 23

9. Solubility of compounds 1 and 5 29

10. ESI-MS and NMR 30

1. Materials

All chemicals were purchased commercially and used without further purification.

2. Instrumentation

Crystallography: Suitable single crystals were selected and mounted onto a rubber loop using Fomblin oil. Single crystal X-ray diffraction data of **1-5** were recorded on a Bruker/ARINAX MD2 diffractometer equipped with a MarCCD-300 detector at beam line station BL17B of Shanghai Synchrotron Radiation Facility (SSRF) at 150 K.

Fourier-transform infrared (FT-IR) spectra: The IR was performed on a Bruker VERTEX 70 infrared spectrometer in the range of 500-4000 cm^{-1}. Intensities are denoted as w = weak, m = medium, s = strong, vs = very strong.

Thermogravimetric Analysis (TGA): Thermogravimetric analysis was performed on a METTLER TOLEDO TG8000 Thermogravimetric Analyzer under nitrogen flow at a typical heating rate of 10 $°C \cdot min^{-1}$ from 30-800 °C.

Element Analyses: Element analyses for P, Mo, K and Na were performed on a Leeman Prodigy Plus inductivity-coupled plasma Inductively Coupled Plasma Optical Emission Spectroscopy (ICP-OES) and C, N and H content were determined by VARIDEL III elemental Analyzer.

UV-vis spectroscopy: The UV-vis was performed on a UV3600 UV-vis spectrophotometer.

Raman spectra: The Raman were recorded on an in Via Reflex laser Raman spectroscopy.

NMR spectroscopy: NMR spectra were recorded on Bruker 400 MHz spectrometers (Avance Neo or AVIII HD; ^{31}P frequency ≈ 162 MHz) equipped with BBFO/SmartProbe. Proton-decoupled ^{31}P spectra were acquired with 2200 scans. Samples were prepared by dissolving pure crystals in D_2O, at approximately 5 mg/0.5 ml.

Electrospray ionization-mass spectrometry (ESI-MS): Both spectra were collected on a Bruker MaXis Impact. Samples were prepared by dissolving pure crystals of **4** and **5** in HPLC CH_3CN, at approximately 1 mg/ml. ESI-MS measurements were carried out at 30 °C. The data

were collected in negative modes. The standard parameters for a medium mass data acquisition were used and the end plate voltage was set to −500 V and the capillary to +4500 V. The collision cell was set to collision energy of −10 eV with a gas flow rate at 38% of maximum and the collision cell RF was set at 2100 Vpp. Transfer time was set to 100 μs.

3. Synthetic procedures for compounds 1-5

Compound 1:

$(NH_4)_6Mo_7O_{24}\cdot 4H_2O$ (494 mg, 0.4 mmol), $NH_2NH_2\cdot 2HCl$ (52 mg, 0.5 mmol) and CH_3COONa (42 mg, 0.5 mmol) were dissolved in 4 mL of deionized water. The resulting dark blue solution was stirred for about 15 min. Afterwards, 1 mL CH_3COOH was added, and the resulting mixture was stirred for additional 30 min until a uniform dark green solution was obtained. Benzylphosphonic acid (80 mg, 0.4 mmol) was then added and the solution was stirred at room temperature for 1 hour. Subsequently, the deep-green solution was further acidified with 6 M HCl to pH 1.9 and stirred for over 30 min. After that, the mixture was transferred to a 10 mL vial sealed with a metallic lid with a rubber septum, which was then heated at 120 °C in an oven for 3 days without any stirring. Dark green crystals suitable for X-ray crystallography were collected and washed with ice water (yield 32% based on Mo). IR (4000-500 cm^{-1}): 3190 (s), 3005 (m), 1616 (s), 1421 (s), 1115 (w), 1054 (w), 968 (s), 881 (w), 775 (m), 687 (w), 632 (m), 531 (m). Elemental analysis % calcd (found) for $C_{160}H_{822}Mo_{136}N_{40}Na_4O_{704}P_{18}$: H 2.93 (2.83), C 6.80 (6.66), N 1.98 (1.96), Na 0.32 (0.19), P 1.97 (1.71), Mo 46.15 (46.58). Mw = 28271.79.

Compound 2:

$(NH_4)_6Mo_7O_{24}\cdot 4H_2O$ (494 mg, 0.4 mmol), $NH_2NH_2\cdot 2HCl$ (52 mg, 0.5 mmol) and CH_3COONa (42 mg, 0.5 mmol) were dissolved in 4 mL of deionized water. The resulting dark blue solution was stirred for about 15 min. Afterwards, 2 mL CH_3COOH was added, and the resulting mixture was stirred for additional 30 min until a uniform dark green solution was obtained. Benzylphosphonic acid (25 mg, 0.15 mmol) was then added and the solution was stirred at room temperature for 1 hour. the deep-green solution was further acidified with 6 M HCl to pH 1.5 and stirred for over 30 min. After that, the mixture was transferred to a 10 mL vial sealed with a metallic lid with a rubber septum, which was then heated at 120 °C in an oven for 3 days

without any stirring. Dark green crystals suitable for X-ray crystallography were collected and washed with ice water (yield 10% based on Mo). IR (4000-500 cm^{-1}): 3193 (s), 2986 (m), 1612 (s), 1510 (s), 1421 (s), 1241 (m), 1112 (m), 1028 (w), 966 (s), 873 (w), 772 (m), 636 (m), 552 (m). Elemental analysis % calcd (found) for $C_{100}H_{518}Mo_{120}N_{20}O_{545}P_{12}$: H 2.30 (2.00), C 5.31 (5.42), N 1.23 (1.18), P 1.64 (1.62), Mo 50.94 (48.88). Mw = 22607.67

Compound 3:

$(NH_4)_6Mo_7O_{24}\cdot4H_2O$ (494 mg, 0.4 mmol), $NH_2NH_2\cdot2HCl$ (52 mg, 0.5 mmol) and CH_3COONa (42 mg, 0.5 mmol) were dissolved in 4 mL of deionized water. The resulting dark blue solution was stirred for about 15 min. Afterwards, 1 mL CH_3COOH was added, and the resulting mixture was stirred for additional 30 min until a uniform dark green solution was obtained. 4-Methoxybenzyl phosphonic acid (88 mg, 0.4 mmol) was then added and the solution was stirred at room temperature for 1 hour. Subsequently, the deep-green solution was further acidified with 6 M HCl to pH 1.6 and stirred for over 30 min. After that, the mixture was transferred to a 10 mL vial sealed with a metallic lid with a rubber septum, which was then heated at 120 °C in an oven for 3 days without any stirring. Dark green crystals suitable for X-ray crystallography were collected and washed with ice water (yield 16% based on Mo). IR (4000-500 cm^{-1}): 3199 (s), 2987 (m), 1610 (s), 1510 (s), 1420 (s), 1242 (m), 1116 (m), 1034 (w), 970 (s), 919 (w), 875 (w), 778 (s), 633 (m), 554 (w). Elemental analysis % calcd (found) for $C_{180}H_{684}Mo_{118}N_{24}Na_2O_{611}P_{20}$: H 2.76 (2.31), C 8.66 (8.10), N 1.34 (1.37), Na 0.18 (0.10), P 2.51 (2.27), Mo 45.37 (46.94). Mw = 24949.77.

Compound 4:

$(NH_4)_6Mo_7O_{24}\cdot4H_2O$ (494 mg, 0.4 mmol), $NH_2NH_2\cdot2HCl$ (52 mg, 0.5 mmol) and CH_3COONa (42 mg, 0.5 mmol) were dissolved in 4 mL of deionized water. The resulting dark blue solution was stirred for about 15 min. Afterwards, 2 mL CH_3COOH was added, and the resulting mixture was stirred for additional 30 min until a uniform dark green solution was obtained. 4-Methoxybenzyl phosphonic acid (50 mg, 0.25 mmol) was then added and the solution was stirred at room temperature for 1 hour. Subsequently, the deep-green solution was further acidified with 6 M HCl to pH 1.8 and stirred for over 30 min. After that, the mixture was transferred to a 10 mL vial sealed with a metallic lid with a rubber septum, which was then

heated at 120 °C in an oven for 3 days without any stirring. Dark green crystals suitable for X-ray crystallography were collected and washed with ice water (yield 20% based on Mo). IR (4000-400 cm^{-1}): 3200 (s), 2990 (m), 1613 (s), 1510 (s), 1420 (s), 1242 (m), 1183 (w), 1114 (m), 1027 (m), 967 (s), 925 (w), 879 (w), 777 (s), 635 (m), 554 (w). Elemental analysis % calcd (found) for $C_{136}H_{564}Mo_{118}N_{24}O_{556}P_{16}$: H 2.44 (2.23), C 7.02 (6.67), N 1.44 (1.45), P 2.13 (1.95), Mo 48.69 (47.14). Mw = 23250.52.

Compound 5:

K_2MoO_4 (476 mg, 2 mmol), $NH_2NH_2{\cdot}2HCl$ (52 mg, 0.5 mmol) and CH_3COONa (42 mg, 0.5 mmol) were dissolved in 4 mL of deionized water. The resulting light green solution was stirred for about 15 min. 4-Hydroxybenzylphosphonic acid (88 mg, 0.4 mmol) was then added and the solution was stirred at room temperature for 1 hour. Subsequently, the brown solution was further acidified with 6 M HCl to pH 1.3 and stirred for over 30 min. After that, the mixture was transferred to a 10 mL vial sealed with a metallic lid with a rubber septum, which was then heated at 120 °C in an oven for 3 days without any stirring. Dark blue crystals suitable for X-ray crystallography were collected and washed with ice water (yield 24% based on Mo).IR (4000-500 cm^{-1}): 3202 (s), 2986 (m), 1613 (s), 1512 (m), 1424 (s), 1238 (w), 1102 (m), 975 (w), 902 (s), 919 (w), 846 (w), 791 (w), 673 (w), 543 (s). Elemental analysis % calcd (found) for $C_{126}H_{704}K_{27}Mo_{157}Na_5O_{803}P_{18}$: H 2.22 (2.26), C 4.74 (5.10), Na 0.35 (0.24), K 3.31 (3.24), P 1.74 (1.78), Mo 47.27 (46.67). Mw = 31861.54.

4. Single-crystal X-ray diffraction analyses

Suitable single crystals were selected and mounted onto a rubber loop using Fomblin oil. Single crystal X-ray diffraction data of **1-5** were recorded on a Bruker/ARINAX MD2 diffractometer equipped with a MarCCD-300 detector at beam line station BL17B of Shanghai Synchrotron Radiation Facility (SSRF) at 150 K with radiation $\lambda = 0.68883$ Å. Data collection and reduction were performed using the Apex 3 software package. Structure solution and refinement were carried out by SHELXT-2019/3[1] and SHELXL-2019/3[2] using Olex2[3] and finalized using WinGX.[4] Most of the non-hydrogen atoms (including those disordered) were anisotropically refined. Crystallographic formulae typically contain many more water molecules in the crystal

lattice than found in the dried sample. Overall, the final refinement statistics were pretty good, and in all cases the structural analysis allows us to unambiguously determine the structures of the compounds. All structure data of compounds **1-5** were deposited at Cambridge Crystallographic Data Center; the data can be obtained via www.ccdc.cam.ac.uk/data_request/cif under deposition numbers CCDC 2505657-2505661.

Structure solution and refinement special details for 1 {Mo_{136}}: one quarter of the {Mo_{136}} cluster was found in the asymmetric unit. Several Mo atoms (Mo24, Mo25, Mo26 and Mo39) within the main cluster framework were modelled as disordered over two positions. The {Mo_2NaL_2} group involving the Na31 atom was well defined, with an average Na-O distance of approximately 2.3 Å. The Mo23 and Mo36 sites in {Mo_3L_2} groups were identified as having major Mo occupancy, accompanied by a minor Na component at low occupancy. Significant disorder was observed in the phenyl groups of organophosphonate (L) ligands. Methyl groups from acetate ligands, either on the cluster or in the solvent region, could not be resolved due to severe disorder. O, OH or H_2O ligands on cluster were assigned based on BVS analysis of oxygen atoms. No H atoms were added to oxygen sites, consistent with the nature of this heavy-metal cluster compound. The same treatment was also applied to the other structures. The SQUEEZE procedure (from PLATON) was employed to estimate the void space and electron counts in the solvent region. The results indicated an accessible volume of 8915 Å^3 and 3129 electrons, corresponding to 40 ammonium cations and approximately 120 additional solvent water molecules per cluster. The reported crystallography formula $C_{160}H_{822}Mo_{136}N_{40}Na_4O_{704}P_{18}$ was determined by combining structure refinement data, SQUEEZE procedure results, and elemental analysis.

Structure solution and refinement special details for 2 {Mo_{120}}: one quarter of the {Mo_{120}} cluster was located in the asymmetric unit. Several Mo atoms (Mo3, Mo7, Mo25) within the main cluster framework were modelled as disordered over two positions. The Mo14 and Mo30 sites in {Mo_3L_2} groups were also modelled as disordered over two positions. Capping {Mo_{11}} group involving Mo32-Mo38 was modelled with 20% occupancy. Significant disorder was observed in the phenyl groups of organophosphonate ligands. The SQUEEZE procedure (from PLATON) was applied to estimate the void space and electron counts in the solvent region. The

results indicated an accessible volume of 6822 Å^3 and 1959 electrons, corresponding to 20 ammonium cations and approximately 70 additional solvent water molecules per cluster. The reported crystallography formula $C_{100}H_{518}Mo_{120}N_{20}O_{545}P_{12}$ was determined by combining structure refinement data, SQUEEZE procedure results, and elemental analysis.

Structure solution and refinement special details for 3 {$Mo_{118}Na_2$}: Half of the {$Mo_{118}Na_2$} cluster was observed in the asymmetric unit. The {Mo_2NaL_2} group involving the Na1 atom was well defined, with an average Na-O distance of approximately 2.3 Å. The {Mo_3L_2} groups containing the Mo7, Mo13, Mo29 and Mo55 sites were clearly resolved with full occupancy. A small ring-shaped Mo cluster comprising Mo61-Mo65 was identified with about 10% occupancy, though it could not fully modelled. Several phenyl groups of organophosphonate ligands exhibit disorder over two positions. The SQUEEZE procedure (from PLATON) was applied to estimate the void space and electron counts in the solvent region. The results indicated an accessible volume of 11146 Å^3 and 3042 electrons, corresponding to 24 ammonium cations and approximately 120 additional solvent water molecules per cluster. The final crystallography formula $C_{180}H_{684}Mo_{118}N_{24}Na_2O_{611}P_{20}$ was determined by combining structure refinement data, SQUEEZE procedure results, and elemental analysis.

Structure solution and refinement special details for 4 {Mo_{118}}: one quarter of the {Mo_{118}} cluster was found in the asymmetric unit. Several Mo atoms (Mo2 and Mo30) within the main cluster framework were modelled as disordered over two positions. The Mo15 site in {Mo_3L_2} group was identified as having 65% occupancy in structure refinement. The SQUEEZE procedure (from PLATON) was applied to estimate the void space and electron counts in the solvent region. The results showed an accessible volume of 14200 Å^3 and 4445 electrons, corresponding to 24 ammonium cations and approximately 190 additional solvent water molecules per cluster. The reported formula $C_{136}H_{564}Mo_{118}N_{24}O_{556}P_{16}$ was determined by combining structure refinement data and elemental analysis.

Structure solution and refinement special details for 5 {Mo_{157}}: a complete {Mo_{157}} cluster was found in the asymmetric unit, comprising a main {Mo_{141}} dodecameric MB wheel and two half-occupied ε-Keggin-based {Mo_{16}} guest clusters. Several Mo atoms (Mo1C, Mo1H, Mo1M, Mo2R, Mo2S, MO2T, Mo2U, Mo2V, Mo2W, Mo2X, Mo2Y and Mo2Z) within the {Mo_{141}}

cluster wheel were modelled as disordered over two positions. The growing {g-Mo_1} sites in all nine {Mo_3L_2} groups were clearly resolved with full occupancy. Two half-occupied ε-Keggin-based {Mo_{16}} guests, each composed of a fully reduced ε-Keggin core {$Mo^V{}_{12}$} and four {Mo^{VI}} add-on units are captured in the dodecameric wheel. High R values of the X-ray structure of **5** can be attributed to the severe disorder of these {Mo_{16}} guest clusters within the framework - not all electron-density residuals in these regions could be fully modelled. Six potassium sits are located between one {Mo_{16}} guest and the {Mo_{141}} wheel framework, with the {Mo_{16}} cluster size and potassium ionic radius fitting the cavity of the dodecameric wheel remarkably well. The second {Mo_{16}} guest cluster connects to the main {Mo_{141}} wheel framework through three {Mo_1} bridges. Minor disorder was observed in the hydroxyphenyl groups of the organophosphonate ligands. The SQUEEZE procedure (from PLATON) was applied to estimate the void space and electron counts in the solvent region, yielding an accessible volume of 55149 Å^3 and 21129 electrons, corresponding to the disordered 26 K+ and 5 Na+ in solvent area and approximately 400 additional solvent water molecules per cluster. The reported formula $C_{126}H_{704}K_{27}Mo_{157}Na_5O_{803}P_{18}$ was determined by combining structure refinement data, and elemental analysis.

5. Crystallographic data and crystal structures of 1-5

Table S1 Crystallographic Details for **1-2**

Compound	**1**	**2**
Empirical formula	$C_{160}H_{822}Mo_{136}N_{40}Na_4O_{704}P_{18}$	$C_{100}H_{518}Mo_{120}N_{20}O_{545}P_{12}$
Formula weight	28271.79	22607.67
Temperature/K	150.0	150.0
Crystal system	monoclinic	monoclinic
Space group	*C2/m*	*C2/m*
a/Å	49.791(5)	37.359(3)
b/Å	33.963(4)	38.692(3)
c/Å	22.584(3)	27.492(2)
α/°	90	90
β/°	111.235(3)	127.088(2)
γ/°	90	90
V/Å^3	35598(7)	31701(4)
Z	2	2
ρ calc /g·cm^{-3}	2.638	2.367
μ(MoKα) /mm^{-1}	2.180	2.325
F(000)	27440.0	21656
2θ range /°	2.88 to 49.294	2.984 to 53.918
Index ranges	$-59 \le h \le 59$,<br>$-41 \le k \le 41$,<br>$-27 \le l \le 27$	$-48 \le h \le 48$,<br>$-50 \le k \le 50$,<br>$-35 \le l \le 36$
Independent reflections	33257 [R_{int} = 0.0657]	37667 [R_{int} = 0.0502]
Data / restraints / parameters	33257/159/1929	37667/244/1740
R_1/wR_2 (I>2σ(I))[a]	R_1 = 0.0897, wR_2 = 0.2087	R_1 = 0.0678, wR_2 = 0.1594
R_1/wR_2 (all data)	R_1 = 0.1017, wR_2 = 0.2244	R_1 = 0.0772, wR_2 = 0.1643
GooF (all data)[b]	1.096	1.086

[a] $R_1 = \Sigma||Fo|-|Fc||/\Sigma|Fo|$; $wR_2 = \{\Sigma w[(Fo)^2-(Fc)^2]^2/\Sigma w[(Fo)^2]^2\}^{1/2}$

[b] $GooF = \{\Sigma w[(Fo)^2-(Fc)^2]^2/(n-p)\}^{1/2}$

Table S2 Crystallographic Details for **3-4**

Compound	**3**	**4**
Empirical formula	$C_{180}H_{684}Mo_{118}N_{24}Na_2O_{611}P_{20}$	$C_{136}H_{564}Mo_{118}N_{24}O_{556}P_{16}$
Formula weight	24949.77	23250.52
Temperature/K	150.0	150.0
Crystal system	monoclinic	monoclinic
Space group	$P2_1/n$	$C2/m$
a/Å	28.825(2)	36.999(4)
bÅ	44.983(3)	44.766(4)
c/Å	29.381(2)	27.184(2)
α/°	90	90
β/°	99.649(2)	127.270(2)
γ/°	90	90
V/Å^3	37558(5)	35831(6)
Z	2	2
ρ calc /g·cm^{-3}	2.206	2.155
μ(MoKα) /mm^{-1}	1.802	1.867
F(000)	24196.0	22384.0
2θ range /°	1.98 to 49.08	1.604 to 49.10
Index ranges	$-34 \leq h \leq 34$, $-54 \leq k \leq 54$, $-35 \leq l \leq 35$	$-44 \leq h \leq 44$, $-53 \leq k \leq 53$, $-32 \leq l \leq 32$
Independent reflections	67891 [R_{int} = 0.0405]	33049 [R_{int} = 0.0485]
Data / restraints / parameters	67891/308/3745	33049/26024/1663
R_1/wR_2 (I>2σ(I))[a]	R_1 = 0.0374, wR_2 = 0.1002	R_1 = 0.0615, wR_2 = 0.1595
R_1/wR_2 (all data)	R_1 = 0.0402, wR_2 = 0.1022	R_1 = 0.0711, wR_2 = 0.1674
GooF (all data)[b]	1.022	1.030

[a]$R_1 = \Sigma||Fo|-|Fc||/\Sigma|Fo|$; $wR_2 = \{\Sigma w[(Fo)^2-(Fc)^2]^2/\Sigma w[(Fo)^2]^2\}^{1/2}$

[b]$GooF = \{\Sigma w[(Fo)^2-(Fc)^2]^2/(n-p)\}^{1/2}$

Table S3 Crystallographic Details for **5**

Compound	**5**
Empirical formula	$C_{126}H_{704}K_{27}Mo_{157}Na_5O_{803}P_{18}$
Formula weight	31861.54
Temperature/K	150.0
Crystal system	monoclinic
Space group	*P*2/*n*
a/Å	36.79(2)
b/Å	50.46(4)
c/Å	59.50(4)
α/°	90
β/°	91.697(13)
γ/°	90
V/Å^3	110411(123)
Z	4
ρ calc /g·cm^{-3}	1.917
μ(MoKα) /mm^{-1}	1.705
F(000)	61264
2θ range /°	1.47 to 48.992
Index ranges	$-43 \leq h \leq 42$, $-60 \leq k \leq 60$, $-71 \leq l \leq 71$
Independent reflections	198466 [$R_{int} = 0.0727$]
Data / restraints / parameters	198466/9522/8368
R_1/wR_2 (I>2σ(I))[a]	$R_1 = 0.1361$, w$R_2 = 0.3769$
R_1/wR_2 (all data)	$R_1 = 0.1484$, w$R_2 = 0.3858$
GooF (all data)[b]	1.044

[a] $R_1 = \sum||Fo|-|Fc||/\sum|Fo|$; $wR_2 = \{\sum w[(Fo)^2-(Fc)^2]^2/\sum w[(Fo)^2]^2\}^{1/2}$

[b] $GooF = \{\sum w[(Fo)^2-(Fc)^2]^2/(n-p)\}^{1/2}$

6. Formula determination

Structural analysis of 1-5

Although the molybdenum cluster architectures are very complex, the general approach to the structural analysis and formula determination is well documented based on giant wheel- and ball-shaped polyoxomolybdates.[5] The structural analysis requires the following lines of evidence/information to allow the assignment of formula and the structural details coupled with

single-crystal X-ray diffraction:

(i) Redox titration to help determine the number of reduced Mo^V centres.

(ii) Bond valence sum analysis to confirm the terminal oxo positions, reduced Mo^V centres and the positions of the hydroxide ligands.

(iii) Elemental analysis of molybdenum, sodium, potassium, phosphorus and C, H, N analysis.

(iv) TGA to estimate the solvent water molecules.

Therefore, the analysis below both presents this data and demonstrates how the structural assignment is consistent with this data.

Redox titrations

The cerimetric titration was carried out using a 0.005 M solution of Ce^{IV} in 0.5 M of sulphuric acid as oxidant which was added dropwise to a solution of compounds (10 mg in 50 mL of H_2O). Compound **1** was selected as a representative example. After addition of 4.10 mL of the oxidant the colour of the solution turned from deep green to colourless along with a characteristic potential jump showed the presence of 60±2 4d electrons which (formally) corresponds to 60 Mo^V centres (theoretical value for 60 e- reduced species: 4.28 mL). The cerimetric titration of **2-4** was performed in the same way as **1**.

Bond valence sum (BVS) analysis

The BVS values of molybdenum and oxygen atoms in **1-5** were calculated using the following equation[6,7]:

$$V_i = \sum \exp(\frac{r_o - r_{ij}}{B})$$

Where B is a constant, r_o is the bond valence parameter for a given atom pair, and r_{ij} is the bond length between atoms i and j observed in the crystal. The parameters for B and r_o for Mo(6+) and Mo(5+) centres were calculated separately using the corresponding parameters listed below.[7]

Parameter list:

Bond	r_o	B
**Mo(+6)-O**	1.903	0.349
**Mo(+5)-O**	1.888	0.314

Table S4. Average Bond valence sum values for the Mo centers in $\{Mo_5O_6\}$ type double cubanes and the μ_3-O atoms of the $\{(\mu_3\text{-}O)_2O_2\}$-type compartments in **1-5**.

Compound	BVS (Mo)	BVS (μ_3-O)
**1**	5.67	1.35
**2**	5.75	1.34
**3**	5.68	1.36
**4**	5.71	1.35
**5**	5.60	1.38

Table S5. Bond valence sum values of Mo^V in **1-5**.

Compound	Atom	BVS	Atom	BVS
**1**	Mo20	5.15	Mo21	4.83
	Mo22	5.02	Mo27	5.08
	Mo29	4.95	Mo30	4.87
	Mo34	5.27	Mo35	4.94
	Mo37	5.05	Mo38	5.17
	Mo39	5.08		
**2**	Mo13	4.86	Mo22	5.00
	Mo19	5.00	Mo23	4.97
	Mo26	4.92		
**3**	Mo2	4.89	Mo25	4.94
	Mo17	4.90	Mo27	4.95
	Mo21	4.95	Mo43	4.90
	Mo52	4.87	Mo57	4.97
	Mo59	4.91	Mo45	4.95
**4**	Mo15	5.12	Mo26	5.03
	Mo16	4.95	Mo27	4.93
	Mo22	4.86		
**5**	Mo20	4.83	Mo38	4.92
	Mo44	4.84	Mo49	4.66
	Mo56	4.69	Mo65	4.96
	Mo68	4.90	Mo74	4.99
	Mo87	4.78	Mo96	4.99
	Mo1G	4.72	Mo1K	5.10
	Mo1O	4.60	Mo2D	5.00
	Mo2I	4.90	Mo2L	4.92
	Mo2M	5.01	Mo2P	4.95

Summary of structure analysis of 1-5

Compound 1: Firstly, the overall reduction state of **1** (60 electrons reduced) was confirmed using two independent techniques (redox titration and bond valence sum analysis), consistent with previous work.[8] A careful analysis of the bond length of Mo-O bonds and bond valence sums reveals 10 singly and 16 doubly protonated oxygen atoms (coordination water). Singly protonated are the 10 equivalent μ_3-O atoms situated in the incomplete double-cubane-type $\{Mo_5O_6\}$. The terminal O atoms with Mo-O bond length larger than 2.2 Å are assigned as coordination water. The other terminal O atoms and bridging O atoms are considered as O^{2-}, as revealed by the BVS. The Mo^V centers (totally 60 Mo^V) are located at (1) $\{Mo_5O_6\}$-type double cubanes as expected (Table S4, 20 electrons reduced), (2) the edge-shared $\{Mo_2\}$ units (Table S5, 20 electrons reduced) and (3) $\{Mo_1\}$ centers (Supplementary Table S5, 10 electrons reduced). As for the $\{Mo_{11}\}$ caps, each contains 5 Mo^V centers according to the BVS calculation (Table S5). Thus it can be seen that there are totally 60 Mo^V centers in **1** theoretically, which is in line with the experimental results with 60 electrons reduced. In this way, we could determine the overall charge of **1a** in **1** as -56. To balance the negative charge of -56, 40 NH_4^+ and 16 H^+ are proposed as counterions based on elemental analysis result and C, H, N analysis. Finally, TGA curve of **1** exhibits a total weight loss of 13.9% from r.t. to 150 °C, which corresponds to ~210 guest water molecules. Taking into consideration of the obtained information from the above calculations along with single-crystal X-ray diffraction, elemental analyses, bond valence sum analysis, TGA and redox titration, it is possible to determine the overall building-block scheme and overall formula for **1** as

$(NH_4)_{40}H_{16}[\{Mo_{11}\}_{10}\{Mo_1\}_4\{Na_1\}_4\{Mo_{11}\}_2(PO_3C_7H_7)_{18}]\cdot ca.210H_2O$

$(NH_4)_{40}H_{16}[(Mo^V_{60}Mo^{VI}_{76}Na_4(OH)_{10}(H_2O)_{16}O_{380}(CH_3COO)_{10}(PO_3C_7H_7)_{18}]\cdot ca.210H_2O$

Compound 2: The formula of **2** is determined in a similar way to **1**. Firstly, the overall reduction state of **2** (52 electrons reduced) was confirmed using two independent techniques (redox titration and bond valence sum analysis). A careful analysis of the bond length of Mo-O bonds and bond valence sums reveals 10 singly and 19 doubly protonated oxygen atoms (coordination water). Accordingly, the overall charge of **2a** in **2** is -42. TGA curve of **2** exhibits a total weight loss of 10.7% from r.t. to 150 °C, which corresponds to ~130 guest water molecules. In summary, the overall building-block scheme and overall formula for **2** is

determined as

$(NH_4)_{20}H_{22}[\{Mo_{11}\}_{10}\{Mo_1\}_6\{Mo_4\}(PO_3C_7H_7)_{12}]\cdot$ca. $130H_2O$

$\equiv (NH_4)_{20}H_{22}[\{Mo^V_{52}Mo^{VI}_{68}(OH)_{10}(H_2O)_{19}O_{334}(CH_3COO)_8(PO_3C_7H_7)_{12}\}]\cdot$ca.$130H_2O$

Compound 3: Firstly, the overall reduction state of **3** (40 electrons reduced) was confirmed using two independent techniques (redox titration and bond valence sum analysis). A careful analysis of the bond length of Mo-O bonds and bond valence sums reveals 10 singly and 18 doubly protonated oxygen atoms (coordination water). The Mo^V centers (totally 40 Mo^V) are located at (1) $\{Mo_5O_6\}$-type double cubanes as expected (Table S4, 20 electrons reduced), and (2) the edge-shared $\{Mo_2\}$ units (Table S5, 20 electrons reduced). Accordingly, the overall charge of **3a** in **3** is -36. TGA curve of **3** exhibits a total weight loss of 11.4% from r.t. to 150 °C, which corresponds to ~155 guest water molecules. In summary, the overall building-block scheme and overall formula for **3** is determined as

$(NH_4)_{24}H_{12}[\{Mo_{11}\}_{10}\{Mo_1\}_8\{Na_1\}_2(PO_3C_7H_6OCH_3)_{20}]\cdot$ca. $155H_2O$

$\equiv (NH_4)_{24}H_{12}[\{Mo^V_{40}Mo^{VI}_{78}Na_2(OH)_{10}(H_2O)_{18}O_{328}(PO_3C_7H_6OCH_3)_{20}\}]\cdot$ca.$155H_2O$

Compound 4: The formula of **4** is determined in a similar way to **3**. Firstly, the overall reduction state of **4** (40 electrons reduced) was confirmed using two independent techniques (redox titration and bond valence sum analysis). A careful analysis of the bond length of Mo-O bonds and bond valence sums reveals 10 singly and 18 doubly protonated oxygen atoms (coordination water). Accordingly, the overall charge of **4a** in **4** is -30. TGA curve of **4** exhibits a total weight loss of 10.5% from r.t. to 150 °C, which corresponds to ~130 guest water molecules. In summary, the overall building block scheme and overall formula for **4** is determined as

$(NH_4)_{24}H_6[\{Mo_{11}\}_{10}\{Mo_1\}_8(PO_3C_7H_6OCH_3)_{16}]\cdot$ca. $130H_2O$

$\equiv (NH_4)_{24}H_6[\{Mo^V_{40}Mo^{VI}_{78}(OH)_{10}(H_2O)_{18}O_{326}(CH_3COO)_4(PO_3C_7H_6OCH_3)_{16}\}]\cdot$ca.$130H_2O$

Compound **5**: Firstly, the overall reduction state of **5** (54 electrons reduced) was confirmed using two independent techniques (redox titration and bond valence sum analysis). A careful analysis of the bond length of Mo-O bonds and bond valence sums reveals 24 singly and 35 doubly protonated oxygen atoms (coordination water). Singly protonated are the 12 equivalent μ_3-O atoms situated in the incomplete double-cubane-type $\{Mo_5O_6\}$ and 12 bridging hydroxyl linkers in ε-Keggin $\{Mo_{16}\}$. The terminal O atoms with Mo-O bond length larger than 2.2 Å

are assigned as coordination water. The other terminal O atoms and bridging O atoms are considered as O^{2-}, as revealed by the BVS. The Mo^{V} centers (totally 54 Mo^{V}) are located at (1) $\{Mo_5O_6\}$-type double cubanes as expected (Table S4, 24 electrons reduced), and (2) the edge-shared $\{Mo_2\}$ units (Table S5, 18 electrons). As for the ε-Keggin $\{Mo_{16}\}$, each contains 12 Mo^{V} centers according to previously published work.[9] Thus it can be seen that there are totally 54 Mo^{V} centers in **5** theoretically, which is in line with the experimental results with 54 electrons reduced. In this way, we could determine the overall charge of **5a** in **5** as -36. To balance the negative charge of -36, 27 K^{+}, 5 Na^{+} and 4 H^{+} are proposed as counterions based on elemental analysis result and C, H, N analysis. Finally, TGA curve of **5** exhibits a total weight loss of 13.3% from r.t. to 150 °C, which corresponds to ~240 guest water molecules. Taking into consideration of the obtained information from the above calculations along with Single-crystal X-ray diffraction, elemental analyses, bond valence sum analysis, TGA and redox titration, it is possible to determine the overall building-block scheme and overall formula for **5** as

$K_{27}Na_5H_4[\{Mo_{11}\}_{12}\{Mo\}_9\{Mo_{16}\}(PO_3C_7H_8OH)_{18}]\cdot ca.240\ H_2O$

$\equiv K_{27}Na_5H_4[\{Mo^{V}_{54}Mo^{VI}_{103}(OH)_{24}(H_2O)_{35}O_{432}(PO_3C_7H_8OH)_{18}\}]\cdot ca.240\ H_2O$

7. Structural descriptions

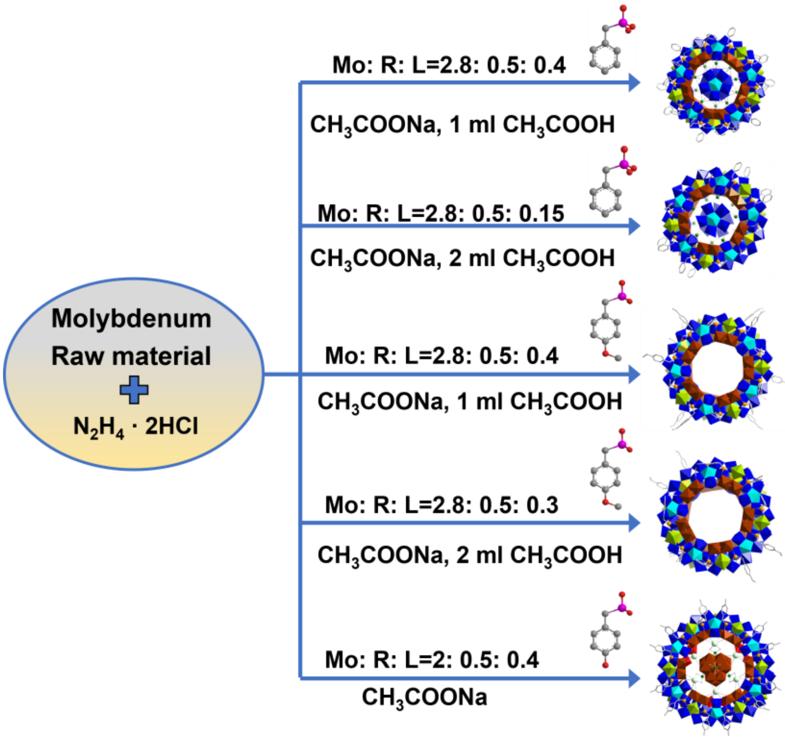

Fig. S1 Summary of the synthesis reactions of **1**-**5**.

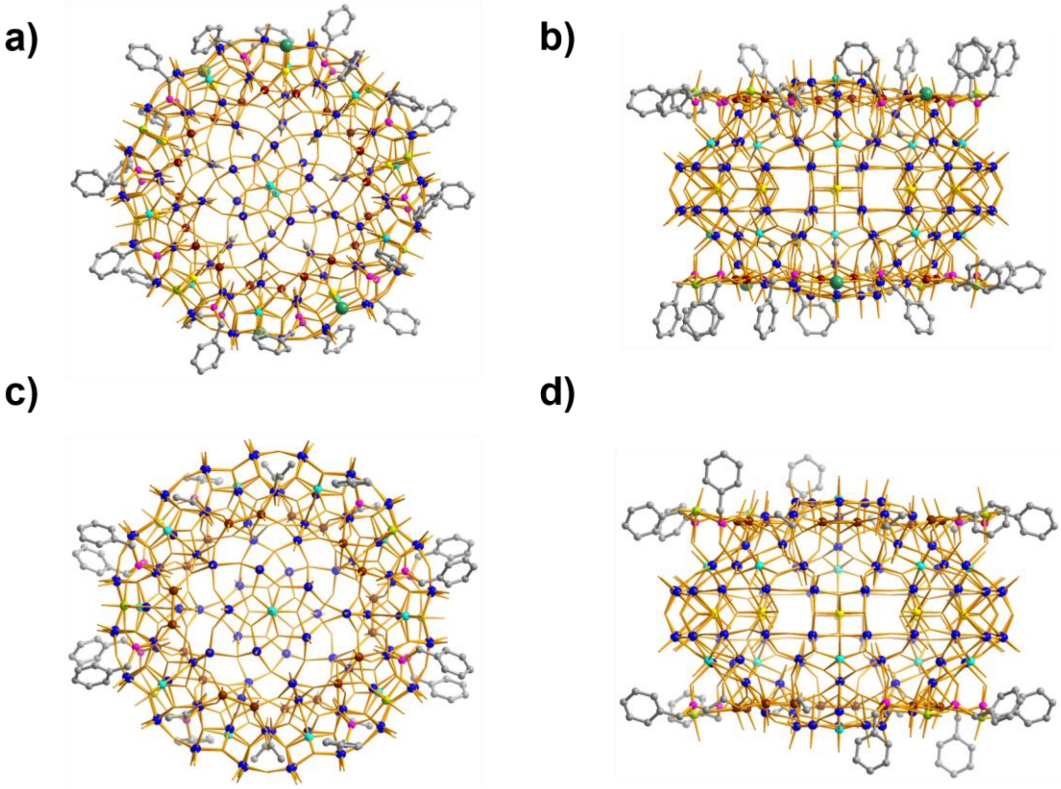

Fig. S2 Ball-and-stick representation of **1a** (a-b) and **2a** (c-d). Color code: Mo blue/dark red/cyan/lime/yellow ball; C, grey ball; O, red ball; P, purple ball; and Na, green ball.

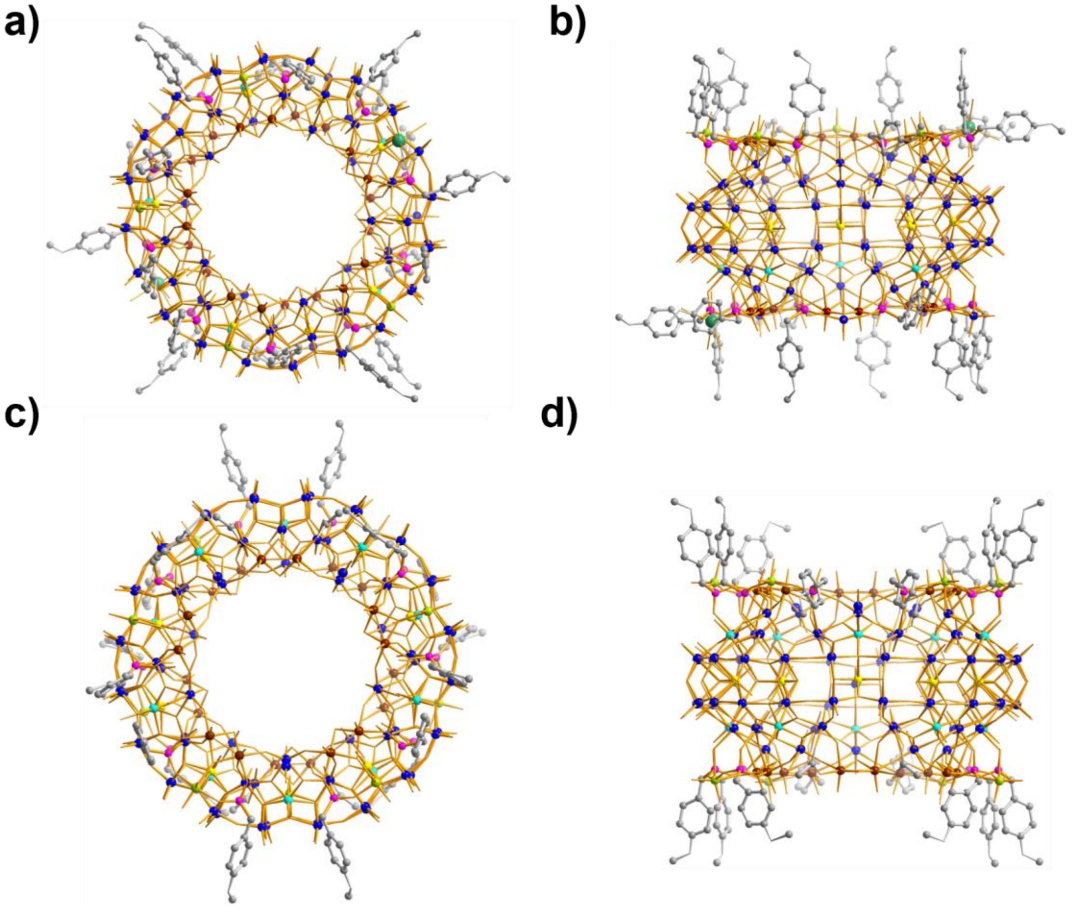

Fig. S3 Ball-and-stick representation of **3a** (a-b) and **4a** (c-d). Color code: Mo blue/dark red/cyan/lime/yellow ball; C, grey ball; O, red ball; P, purple ball; and Na, green ball.

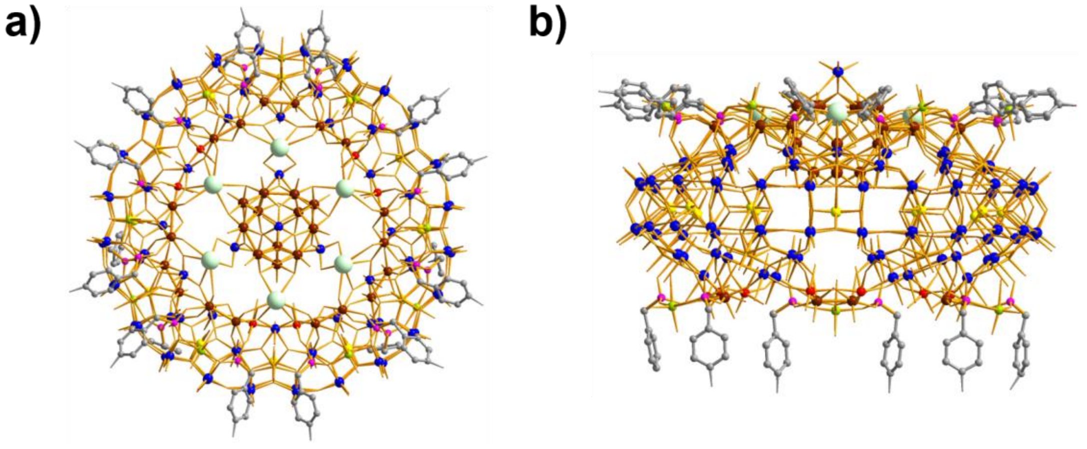

Fig. S4 Ball-and-stick representation of **5a** (a-b). Color code: Mo blue/ dark red/red/cyan/lime/yellow ball; C, grey ball; O, red ball; P, purple ball; and K, light green ball.

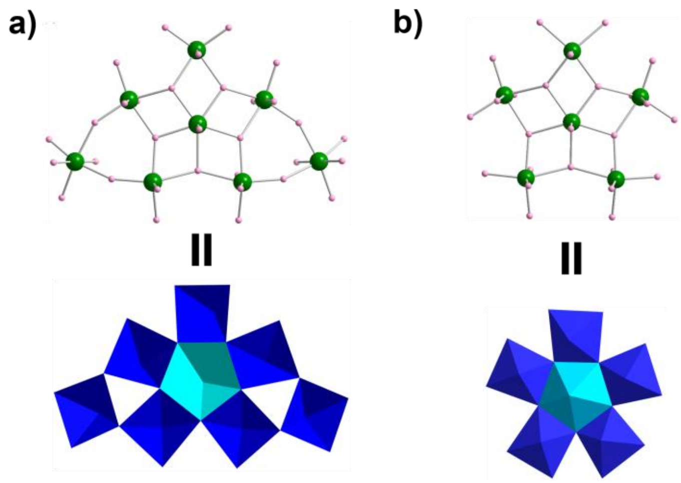

Fig. S5 Combined polyhedral/ball and stick representation of {Mo_8} (a) and pentagon {Mo_6} (b). Color code: Mo, green ball; O, rose ball. {Mo_8} and {Mo_6}, blue polyhedron pentagonal units with cyan polyhedron center.

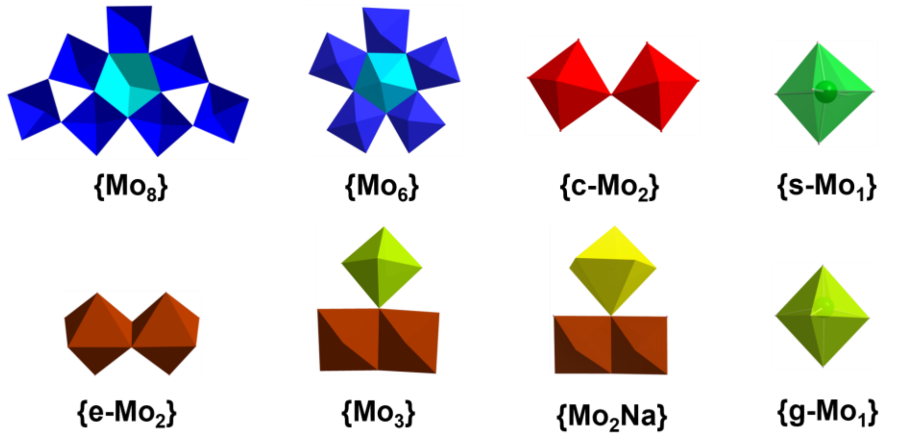

Fig. S6 Polyhedral representations of the structural building blocks constituting the compounds. Top: {Mo_8}, {Mo_6}, {c-Mo_2}, and {s-Mo_1}; Bottom: {e-Mo_2}, {Mo_3}, {Mo_2Na}, and {g-Mo_1}.

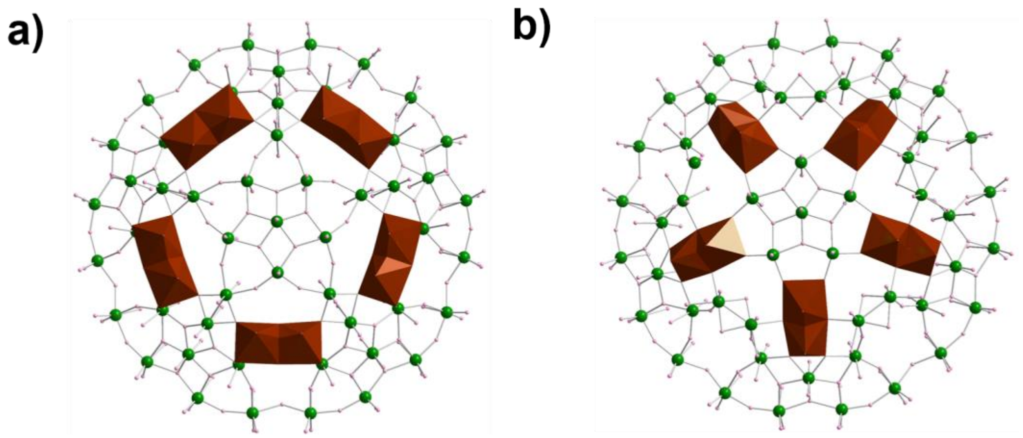

Fig. S7 Combined polyhedral/ball and stick representation of {Mo_{63}} highlights the rim-{e-Mo_2} (a) and beam-{e-Mo_2} (b). Color code: Mo, green ball; O, rose ball; and {e-Mo_2}, dark red polyhedron.

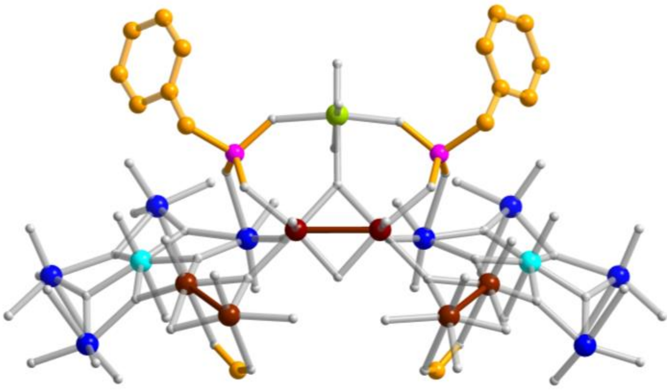

Fig. S8 The binding modes of $PO_3C_7H_7^{2-}$ (a) and CH_3COO^- (b) ligands in **1a**. The CH_3COO^- groups and $PO_3C_7H_7^{2-}$ are shown in the light orange ball-and-stick modes. Color scheme: Mo^{VI} blue/cyan, Mo^{V} dark red; and O grey. The CH_3 groups of the acetate ligands were not found due to too heavy disorders in the single crystal structure analysis.

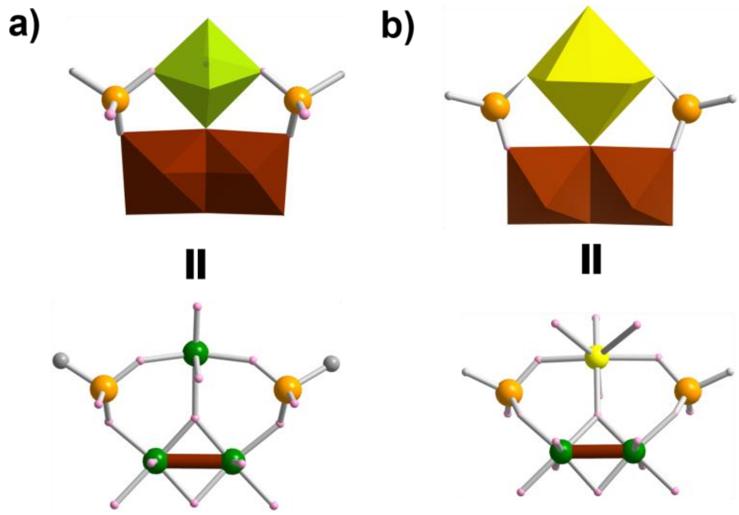

Fig. S9 Combined polyhedral/ball-and-stick representation of $\{Mo_3L_2\}$ and $\{Mo_2NaL_2\}$. Color scheme: Mo, green ball; P, light orange ball; O, rose ball; Na, yellow ball/polyhedron; and {e-Mo_2}, dark red polyhedron.

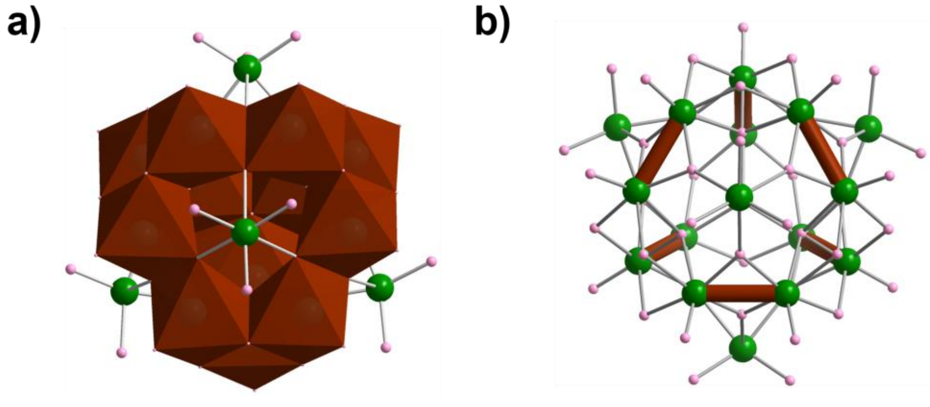

Fig. S10 View of polyhedral and ball-and-stick representation of {$\mathbf{Mo_{16}}$}. Color code: Mo, green ball; and O, rose ball.

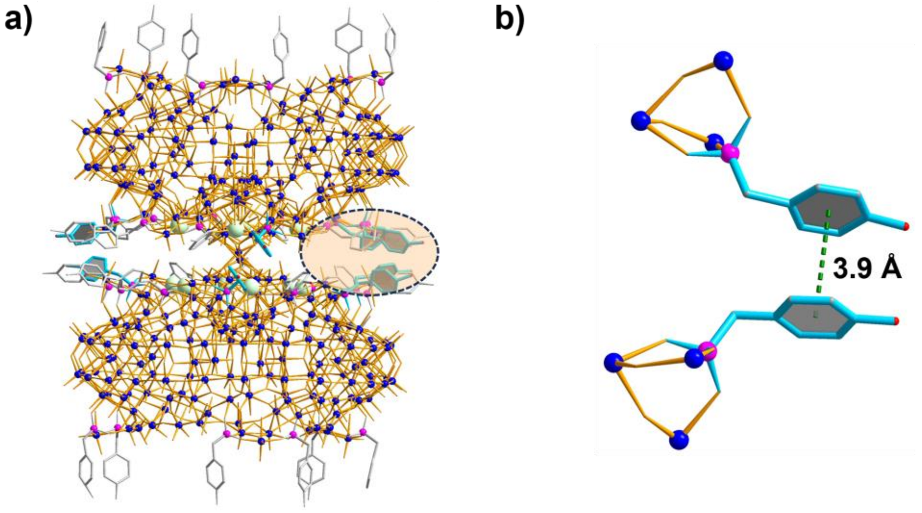

Fig. S11 (a) Dimeric assembly of two adjacent {Mo_{157}} clusters via π–π stacking of organophosphate ligands; (b) enlarged view of the π–π stacking between the aromatic groups. Color code: Mo, blue ball; P, purple ball; and O, red ball. The $PO_3C_7H_6OH^{2-}$ are shown in the blue ball-and-stick modes.

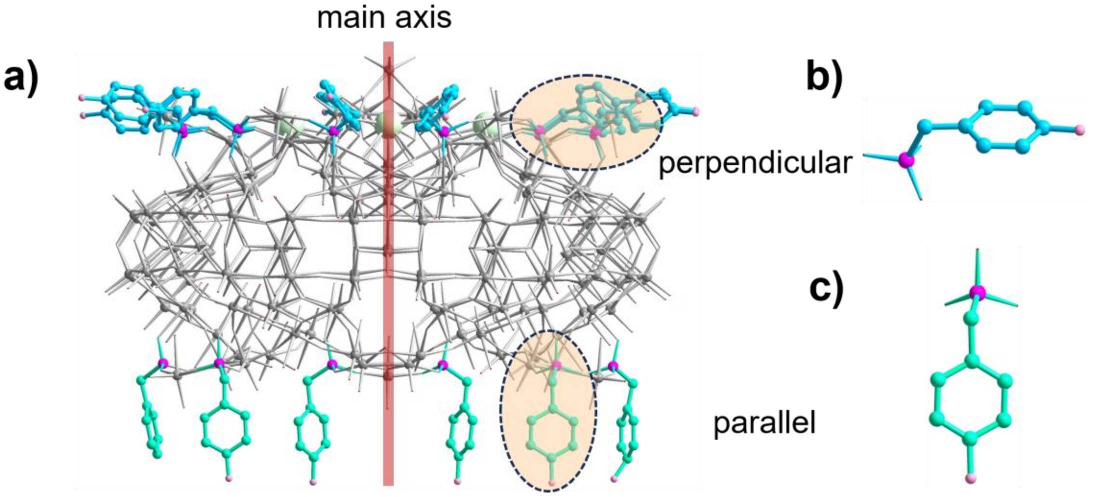

Fig. S12. a) View of ball-and-stick representation of {$\mathbf{Mo_{157}}$} **5a**; b) and c) highlights the orientations of the PhOH groups, perpendicular or parallel to the cluster main axis. Color code: Mo, grey ball; P, purple ball; and O, rose ball. The $PO_3C_7H_6OH^{2-}$ ligands are shown in the cyan and blue ball-and-stick modes.

8. Spectral characterization of 1-5

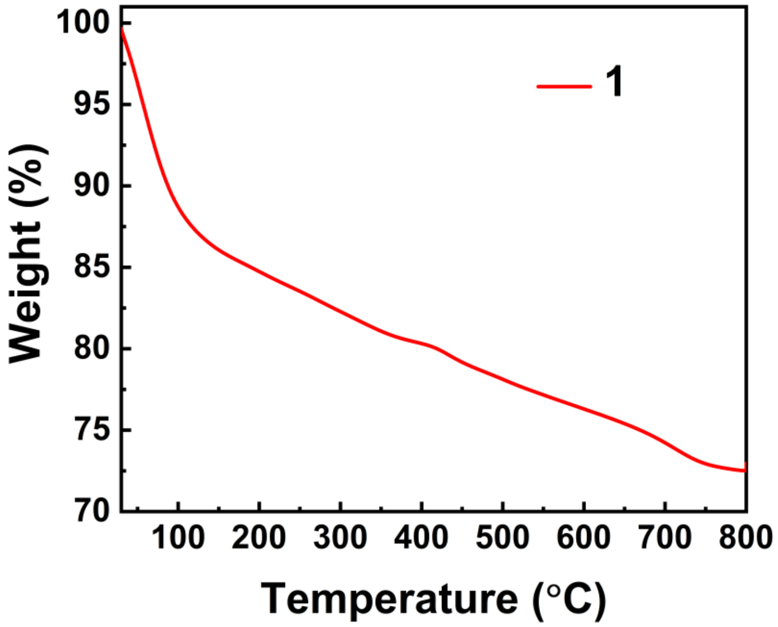

Fig. S13 TGA curve of **1**. 13.9% weight loss between r.t. to 150 °C corresponds to ~210 crystalline H_2O.

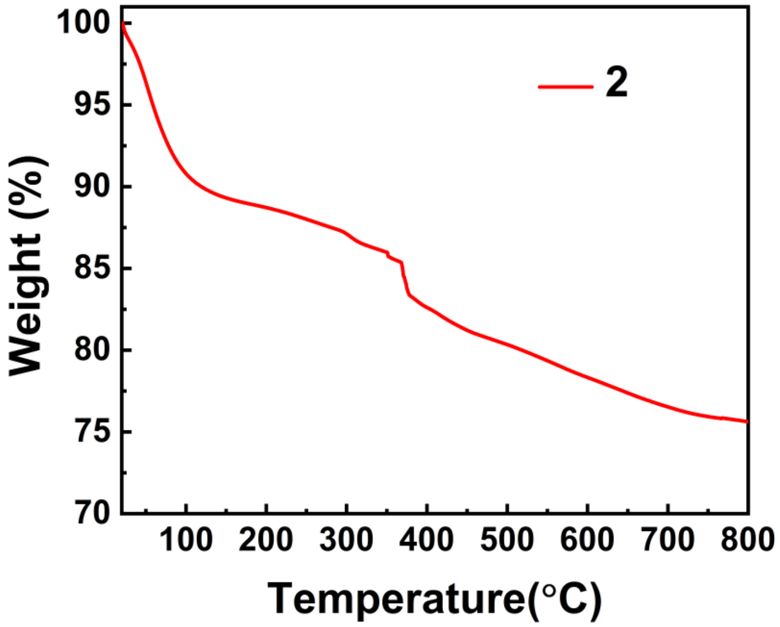

Fig. S14 TGA curve of **2**. 10.7% weight loss between r.t. to 150 °C corresponds to ~130 crystalline H_2O.

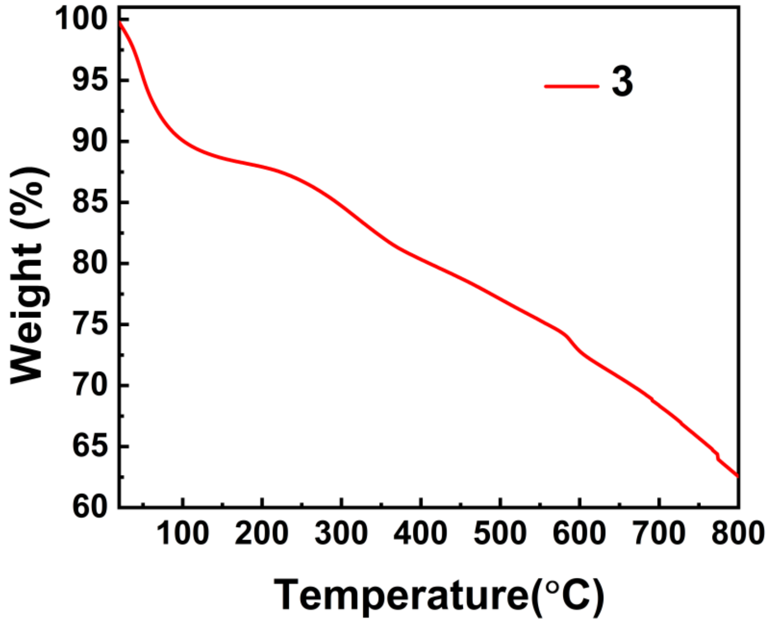

Fig. S15 TGA curve of **3**. 11.4% weight loss between r.t. to 150 °C corresponds to ~155 crystalline H_2O.

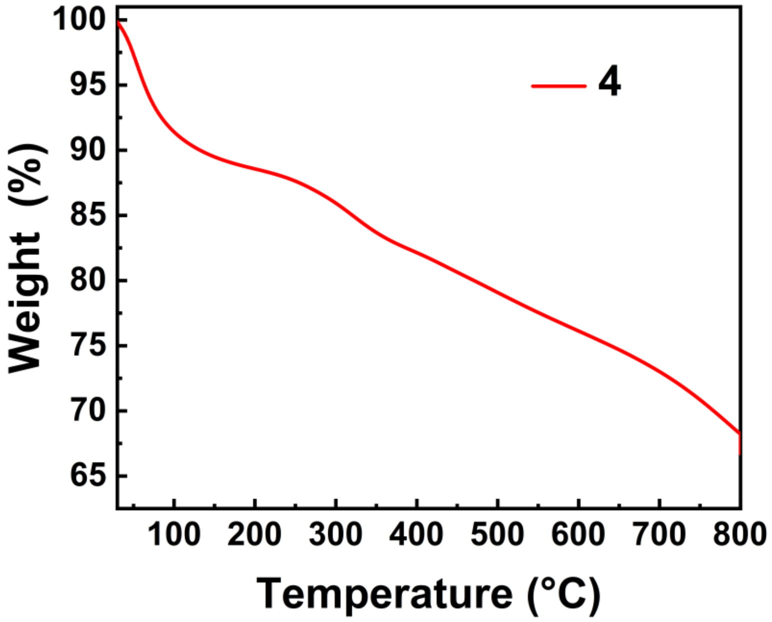

Fig. S16 TGA curve of **4**. 10.5% weight loss between r.t. to 150 °C corresponds to ~130 crystalline H_2O.

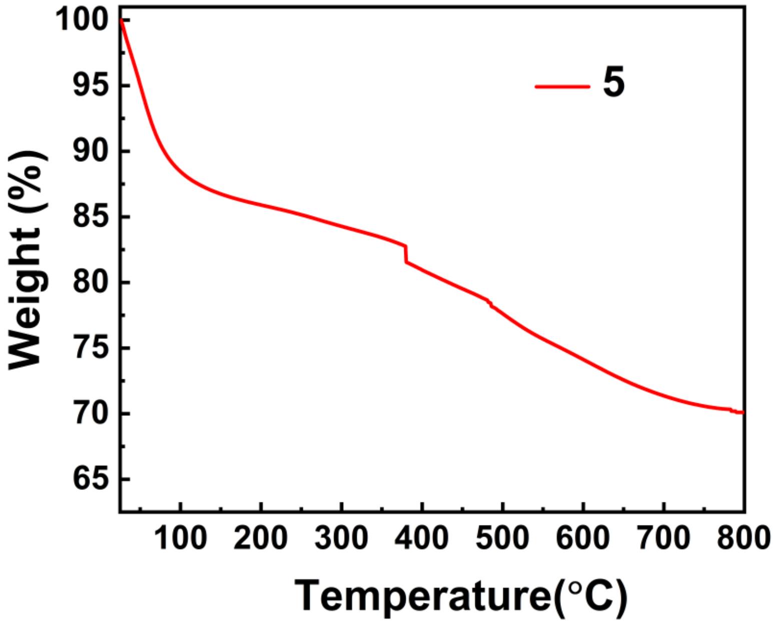

Fig. S17 TGA curve of **5**. 13.3% weight loss between r.t. to 150 °C corresponds to ~240 crystalline H_2O.

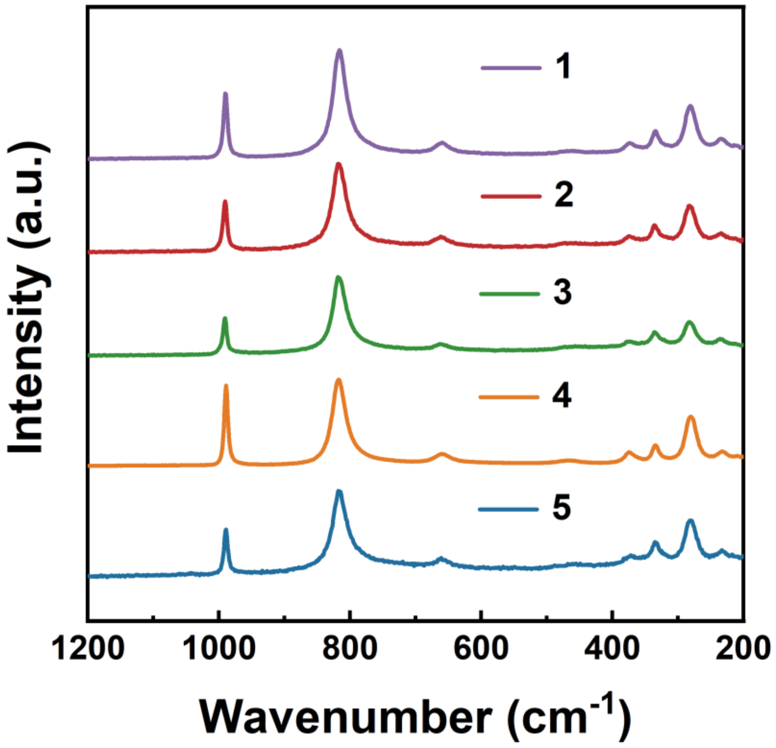

Fig. S18 Raman spectra of **1-5**.

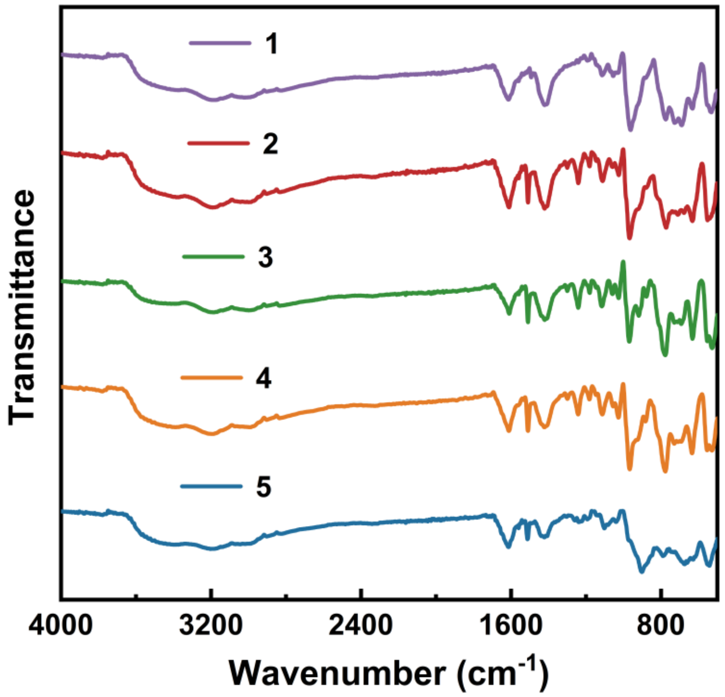

Fig. S19 FT-IR spectra of **1-5**.

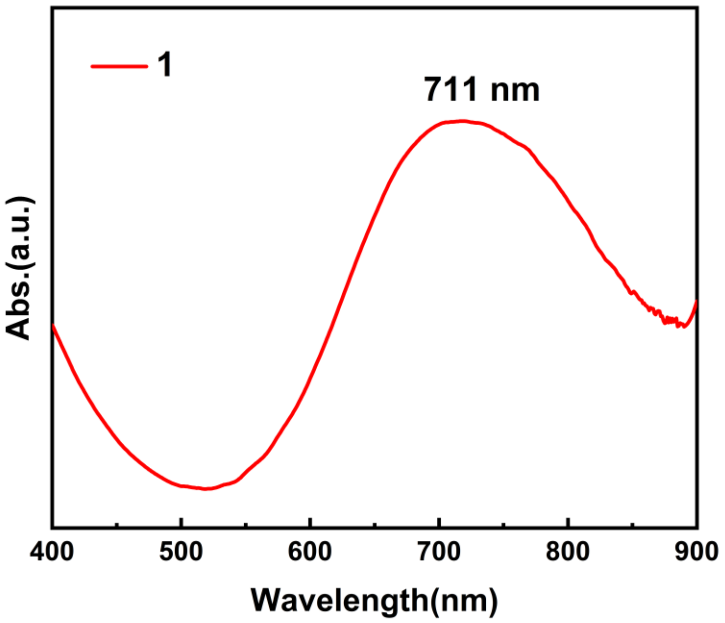

Fig. S20 UV-vis spectrum of **1**.

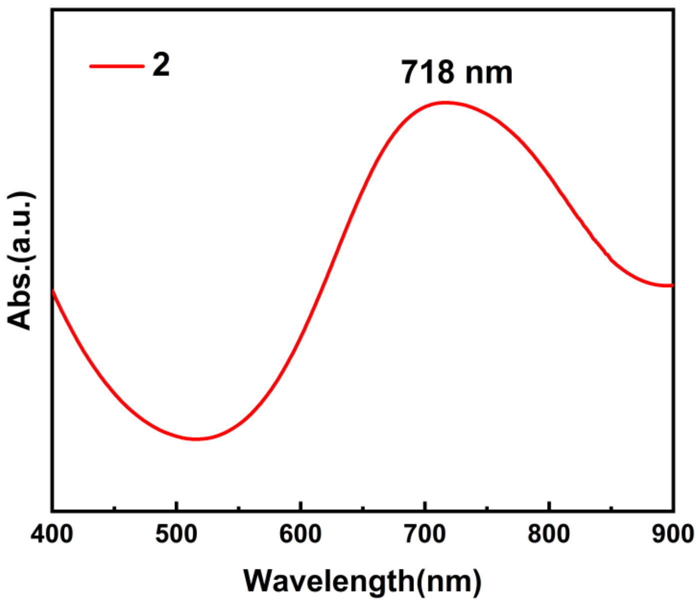

Fig. S21 UV-vis spectrum of **2**.

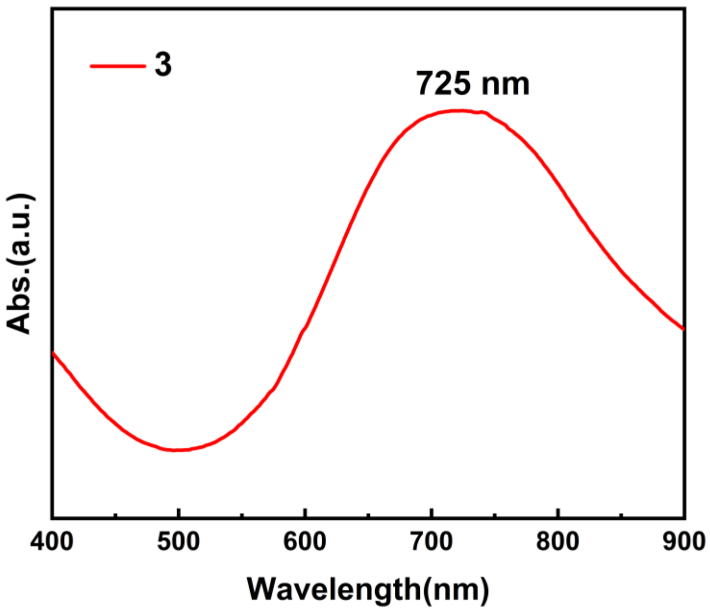

Fig. S22 UV-vis spectrum of **3**.

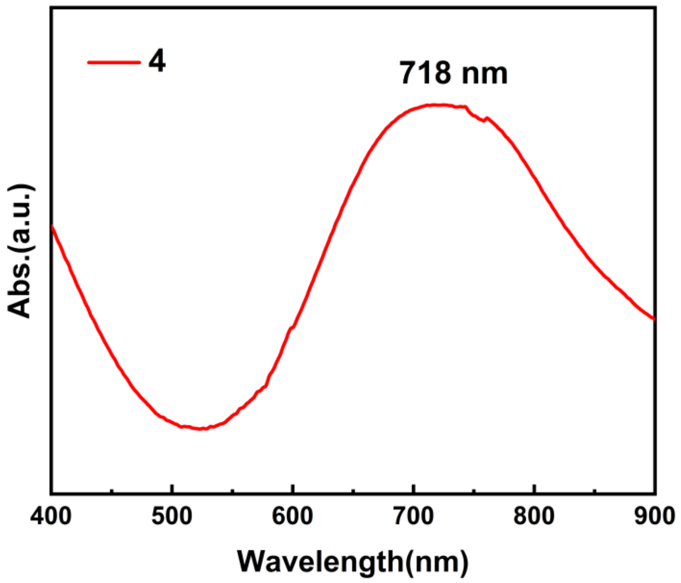

Fig. S23 UV-vis spectrum of **4**.

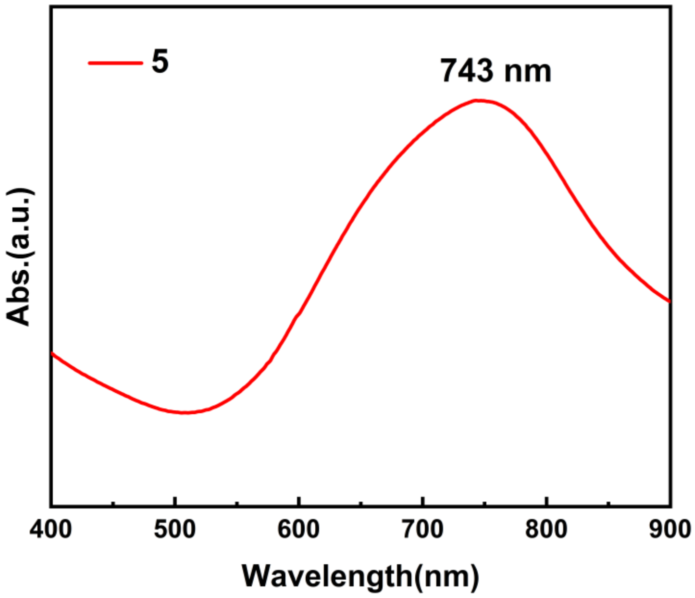

Fig. S24 UV-vis spectrum of **5**.

9. Solubility of compounds 1 and 5

To evaluate solubility in organic solvents, compounds **1** and **5** were selected as representative examples. To compare with traditional molybdenum blue species, {Mo_{154}} was used as a reference compound. Saturated solutions of both compounds were prepared, and equal volumes of clear supernatant were taken. After solvent evaporation, the weight of the residual solid was measured, providing a quantitative of measure solubility.

Table S6. Solubility of {Mo_{136}}, {Mo_{157}} and {Mo_{154}} in different organic solvents.

Compound	Solvent	Solubility
**Mo_{136}**	isopropanol	1.25 g/100 mL
**Mo_{157}**	isopropanol	1.60 g/100 mL
**Mo_{154}**	isopropanol	0.7 g/100 mL
**Mo_{136}**	acetonitrile	0.96 g/100 mL
**Mo_{157}**	acetonitrile	3.2 g/100 mL
**Mo_{154}**	acetonitrile	2.1 g/100 mL
**Mo_{136}**	N,N-dimethylformamide	1.3 g/100 mL
**Mo_{157}**	N,N-dimethylformamide	1.67 g/100 mL
**Mo_{154}**	N,N-dimethylformamide	Not detected
**Mo_{136}**	dichloromethane	Not detected
**Mo_{157}**	dichloromethane	Not detected
**Mo_{154}**	dichloromethane	Not detected

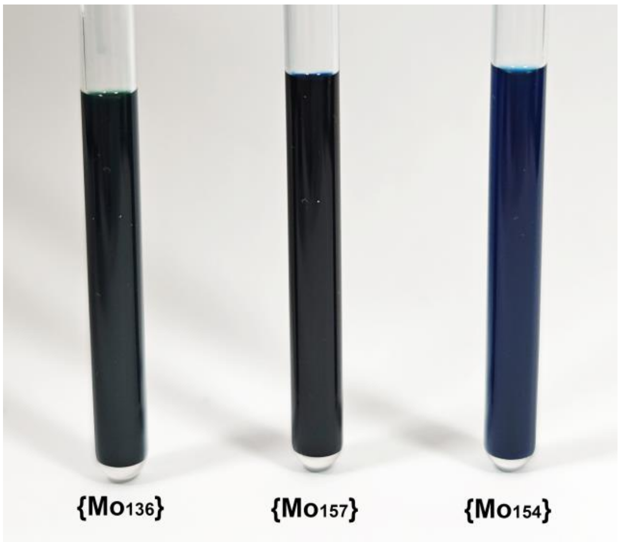

Fig. S25 Color comparison of saturated {Mo_{136}}, {Mo_{157}} and {Mo_{154}} dissolved in isopropanol.

10. ESI-MS and NMR

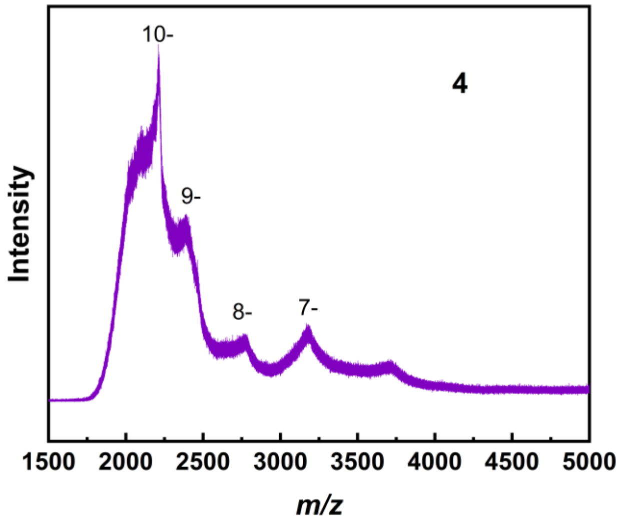

Fig. S26 ESI-MS spectrum of **4** in a CH_3CN solution.

Table S7. Assignment of peaks in negative mode Mass spectrum of compound **4**.

m/z	Charge	Molecular mass	Envelop Assignment
2209.62	-10	22096.2	$\{Mo_{118}O_{354}H_{46}(CH_3COO)_4(PO_3C_7H_6OCH_3)_{16}(NH_4)_{20}(H_2O)_{70}\}^{10-}$
2399.9	-9	21599.1	$\{Mo_{118}O_{354}H_{46}(CH_3COO)_4(PO_3C_7H_6OCH_3)_{16}(NH_4)_{21}(H_2O)_{42}\}^{9-}$
2773.4	-8	22187.2	$\{Mo_{118}O_{354}H_{46}(CH_3COO)_4(PO_3C_7H_6OCH_3)_{16}(NH_4)_{22}(H_2O)_{73}\}^{8-}$
3176.35	-7	22234.45	$\{Mo_{118}O_{354}H_{46}(CH_3COO)_4(PO_3C_7H_6OCH_3)_{16}(NH_4)_{23}(H_2O)_{75}\}^{7-}$
3711.44	-6	22268.64	$\{Mo_{118}O_{354}H_{46}(CH_3COO)_4(PO_3C_7H_6OCH_3)_{16}(NH_4)_{24}(H_2O)_{75}\}^{6-}$

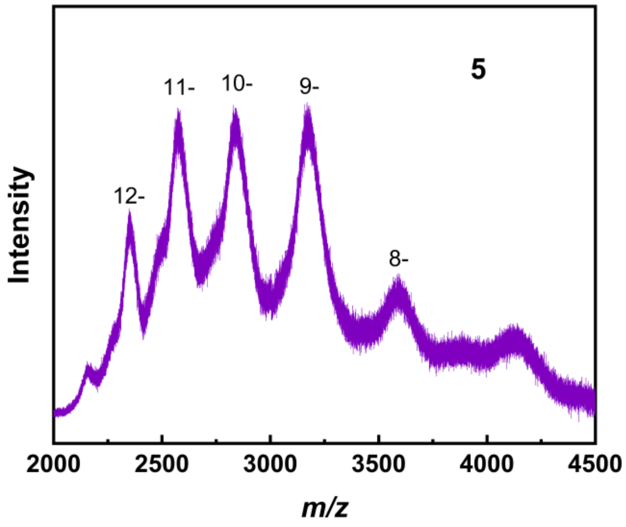

Fig. S27 ESI-MS spectrum of **5** in a CH_3CN solution.

Table S8. Assignment of peaks in negative mode Mass spectrum of compound **5**.

m/z	Charge	Molecular mass	Envelop Assignment
2340.0	-12	28080.0	$\{Mo_{157}O_{456}H_{24}(H_2O)_{35}(PO_3C_7H_8OH)_{18}K_{24}(H_2O)_{41}\}^{12-}$
2572.3	-11	28295.3	$\{Mo_{157}O_{456}H_{24}(H_2O)_{35}(PO_3C_7H_8OH)_{18}K_{25}(H_2O)_{51}\}^{11-}$
2829.0	-10	28290.0	$\{Mo_{157}O_{456}H_{24}(H_2O)_{35}(PO_3C_7H_8OH)_{18}K_{26}(H_2O)_{48}\}^{10-}$
3164.9	-9	28484.1	$\{Mo_{157}O_{456}H_{24}(H_2O)_{35}(PO_3C_7H_8OH)_{18}K_{27}(H_2O)_{57}\}^{9-}$
3573.5	-8	28588.0	$\{Mo_{157}O_{456}H_{24}(H_2O)_{35}(PO_3C_7H_8OH)_{18}K_{27}Na(H_2O)_{62}\}^{8-}$

The envelopes encompass a range of different cation-anion compositions i.e. different combinations of H^+, K^+, Na^+ and solvent molecules is possible, reflected by the broad m/z distribution in the peaks, however the assignment of the anion cluster is consistently linked via the charge-progression analysis.

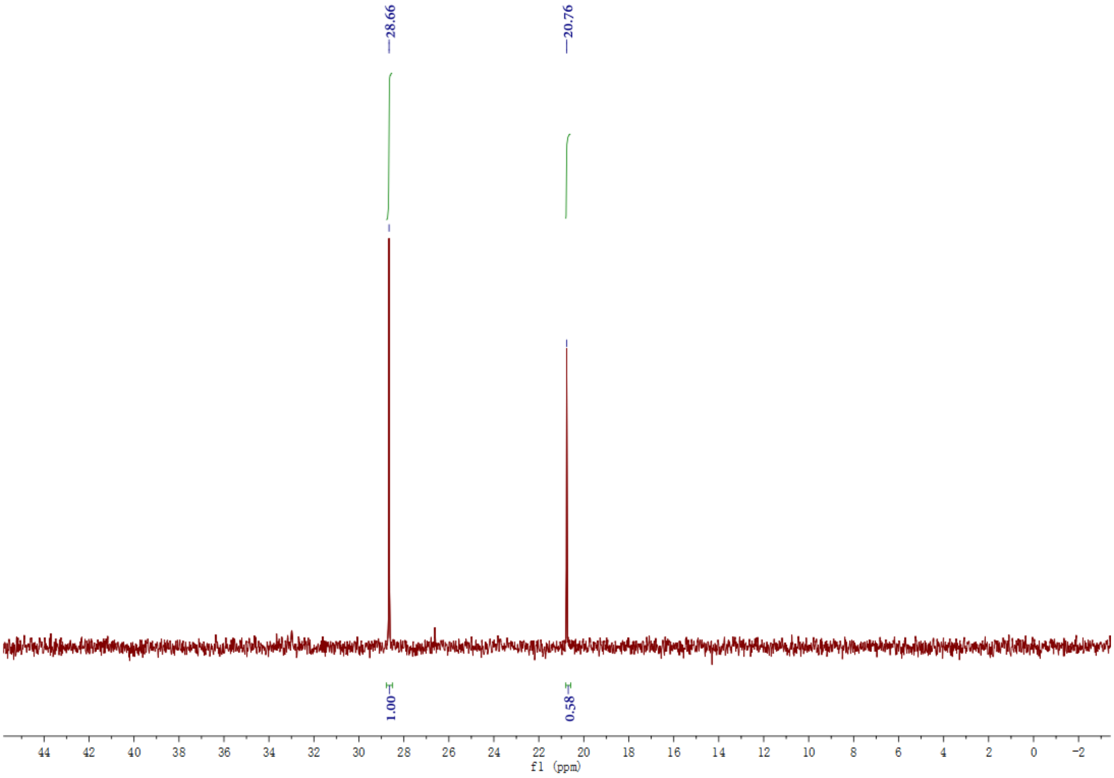

Fig. S28 ^{31}P NMR spectrum of **1**.

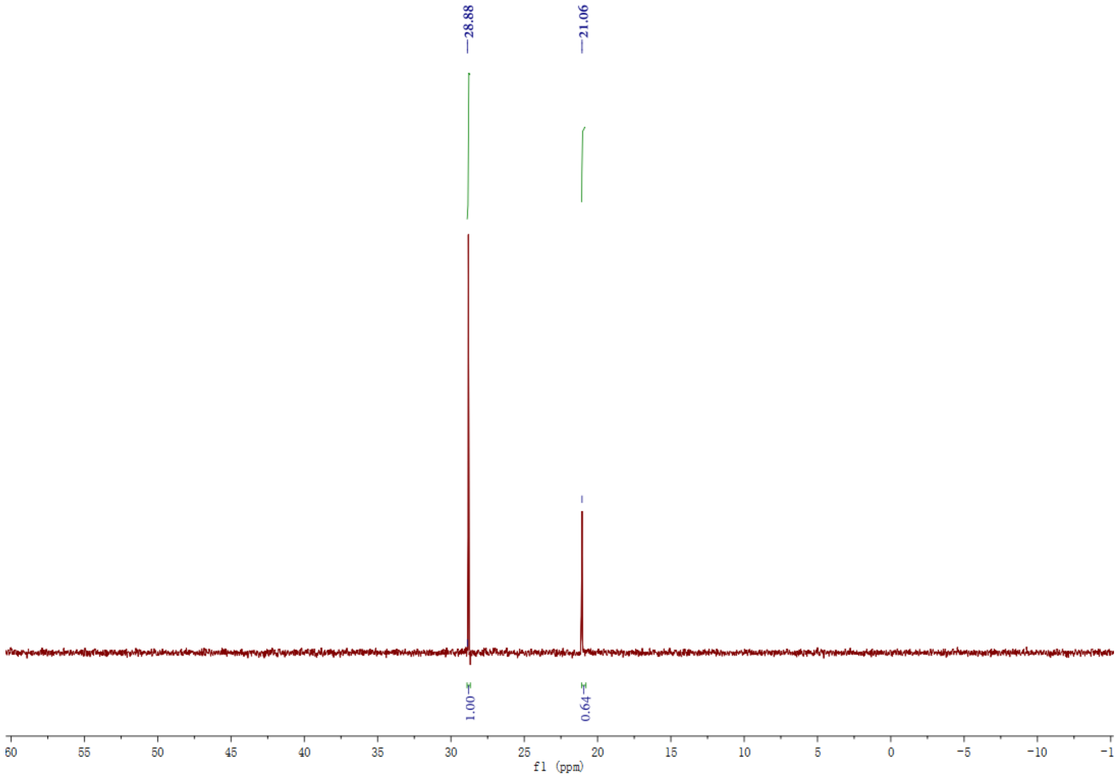

Fig. S29 ^{31}P NMR spectrum of **3**.

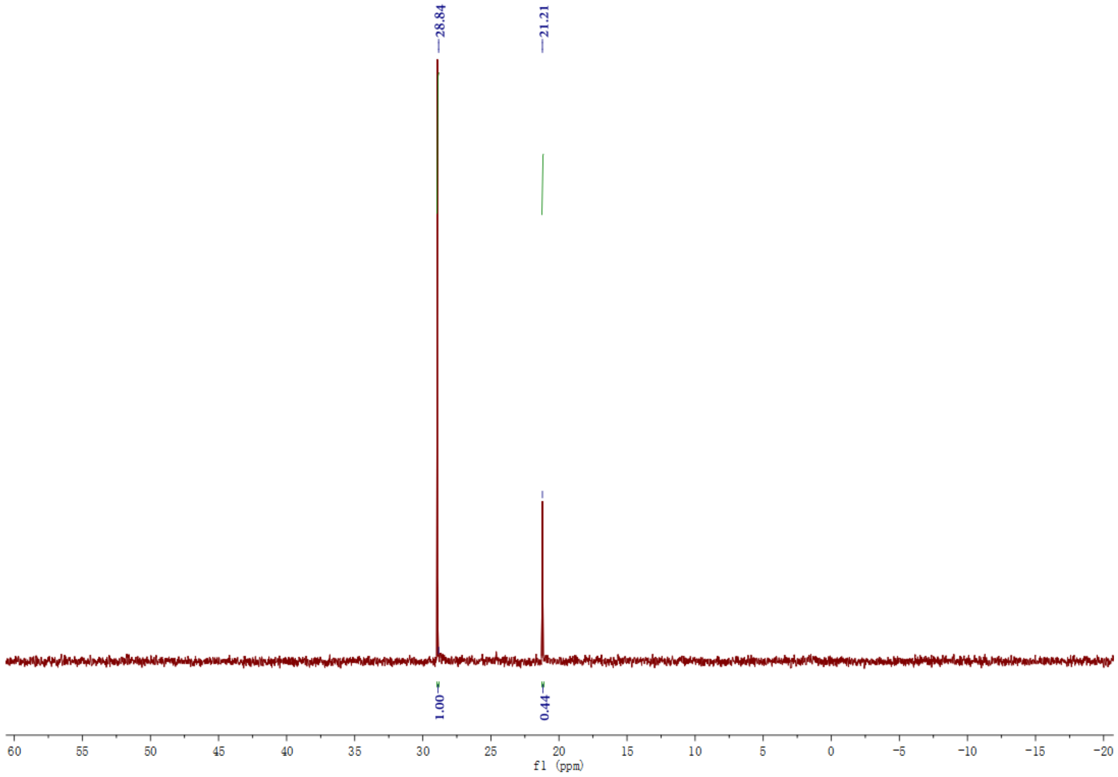

Fig. S30 ^{31}P NMR spectrum of **5**.

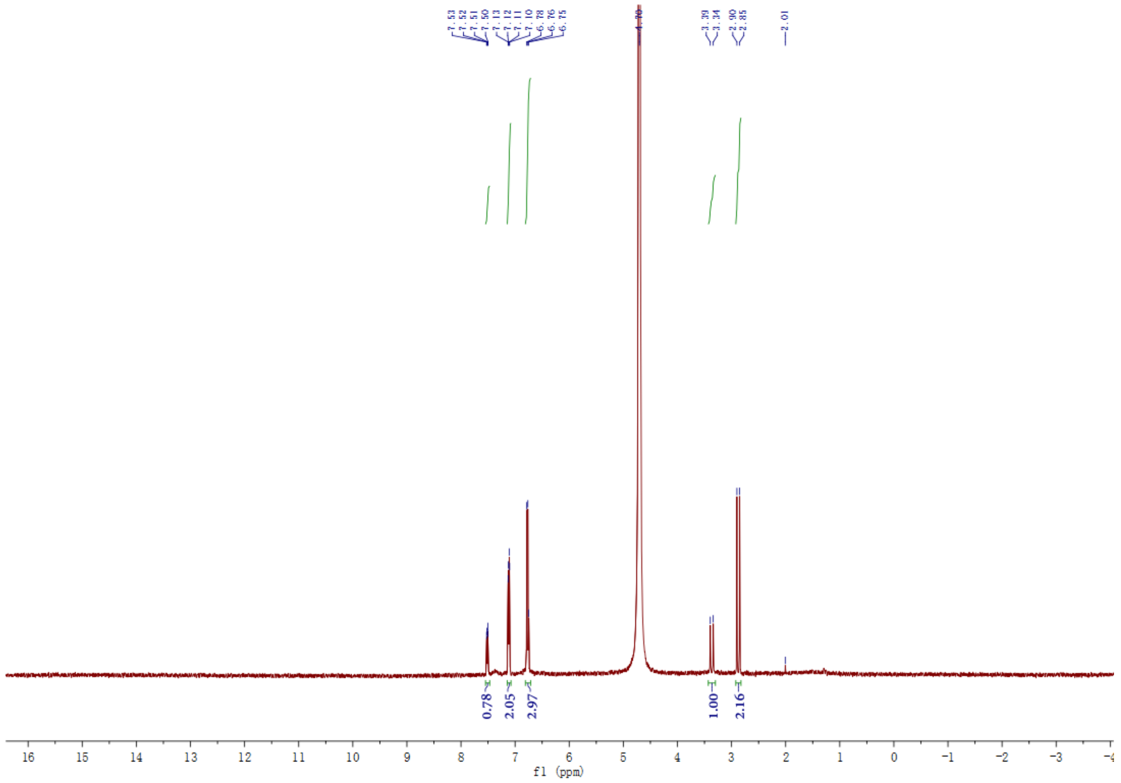

Fig. S31 ^{1}H NMR spectrum of **5**.

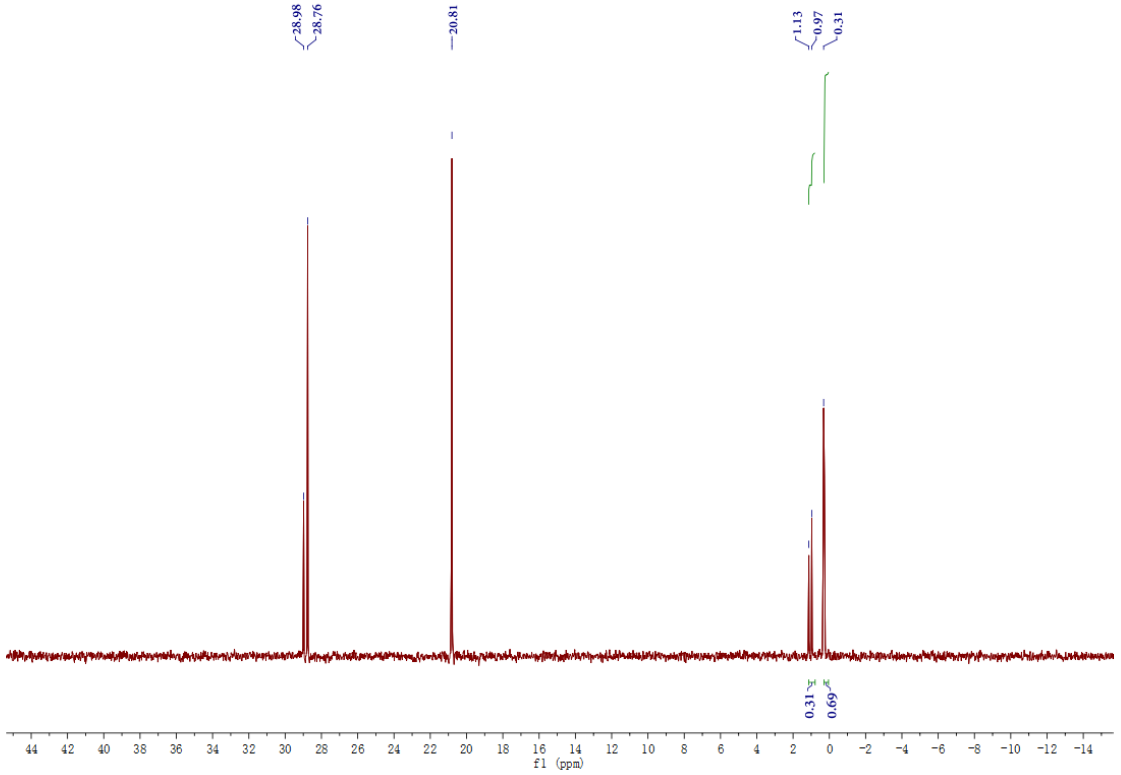

Fig. S32 ^{31}P NMR spectrum of **5a** after the addition of 3 equiv of guanosine monophosphate (GMP).

11. References

(1) Sheldrick, G. M., SHELXT - Integrated space-group and crystal-structure determination. *Acta Crystallogr. Sect. A: Found. Crystallogr.* **2015**, *71*, 3-8.

(2) Sheldrick, G. M., Crystal structure refinement with SHELXL. *Acta Crystallogr. Sect. C: Cryst. Struct. Commun.* **2015**, *71*, 3-8.

(3) Dolomanov, O. V.; Bourhis, L. J.; Gildea, R. J.; Howard, J. A. K.; Puschmann, H., OLEX2: a complete structure solution, refinement and analysis program. *J. Appl. Crystallogr.* **2009**, *42*, 339-341.

(4) Farrugia, L. J., WinGX suite for small-molecule single-crystal crystallography. *J. Appl. Crystallogr.* **1999**, *32*, 837-838.

(5) Müller, A.; Krickemeyer, E.; Bögge, H.; Schmidtmann, M.; Beugholt, C.; Das, S. K.; Peters, F., *Chem. Eur. J.* **1999**, *5*, 1496-1502; b) Müller, A.; Serain, C., *Acc. Chem. Res.* **2000**, *33*, 2-10.

(6) Brese, N. E.; O'Keeffe, M. *Acta Crystallographica Sect. B* **1991**, *47*, 192-197.

(7) a) Gagne, O. C. ; Hawthorne, F. C. *Acta Crystallogr. Sect. B* **2015**, *71*, 562-578. b) Brown, D. I.; Altermatt, D. *Acta Crystallogr. Sect. B* **1985**, *41*, 244-247.

(8) Li, X.-X.; Li, C.-H.; Hou, M.-J.; Zhu, B.; Chen, W.-C.; Sun, C.-Y.; Yuan, Y.; Guan, W.; Qin, C.; Shao, K.-Z.; Wang, X.-L.; Su, Z.-M. Ce-Mediated Molecular Tailoring on Gigantic Polyoxometalate $\{Mo_{132}\}$ into Half-Closed $\{Ce_{11}Mo_{96}\}$ for High Proton Conduction. *Nat. Commun.* **2023**, *14*, 5025.

(9) Khan, M. I.; Müller, A.; Dillinger, S.; Bögge, H.; Chen, Q.; Zubieta, J. Cation Inclusion within the Mixed-Valence Polyanion Cluster $[(Mo^{VI}O_3)_4Mo_{12}O_{28}(OH)_{12}]^{8-}$: Syntheses and Structures of $(NH_4)_7[NaMo_{16}(OH)_{12}O_{40}]\cdot 4H_2O$ and $(Me_2NH_2)_6[H_2Mo_{16}(OH)_{12}O_{40}]$. *Angew. Chem. Int. Ed.* **1993**, *32*, 1780–1782.
